# The Microscopic Derivation and Well-Posedness of the Stochastic Keller–Segel Equation

**DOI:** 10.1007/s00332-020-09661-6

**Published:** 2020-12-18

**Authors:** Hui Huang, Jinniao Qiu

**Affiliations:** 1 Department of Mathematics, Technische Universität München, Boltzmannstr. 3, 85748 Garching, Germany; 2 Department of Mathematics and Statistics, University of Calgary, 2500 University Drive NW, Calgary, AB T2N 1N4 Canada

**Keywords:** Chemotaxis, Propagation of chaos, Bessel potential, Stochastic partial differential equation, 65M75, 35K55, 60H30, 60J7

## Abstract

In this paper, we propose and study a stochastic aggregation–diffusion equation of the Keller–Segel (KS) type for modeling the chemotaxis in dimensions $d=2,3$. Unlike the classical deterministic KS system, which only allows for idiosyncratic noises, the stochastic KS equation is derived from an interacting particle system subject to both idiosyncratic and common noises. Both the unique existence of solutions to the stochastic KS equation and the mean-field limit result are addressed.

## Introduction

Many bacteria, such as *Escherichia coli*, Rhodobacter sphaeroides and Bacillus subtilus, are able to direct their movements according to the surrounding environment by a biased random walk. For example, bacteria try to swim toward the highest concentration of nutrition or to flee from poisons. In biology, this phenomenon is called *chemotaxis*, which describes the directed movement of cells and organisms in response to chemical gradients. Chemotaxis is also observed in other biological fields, for instance the movement of sperm toward the egg during fertilization, the migration of neurons or lymphocytes, and inflammatory processes.

Mathematically, one of the most classical models for studying chemotaxis is the Keller–Segel (KS) equation that was originally proposed in Keller and Segel (1970) to characterize the aggregation of the slime mold amoebae. The classical parabolic-elliptic type KS equation is of the following form:

1.1 $$\begin{aligned} {\left\{ \begin{array}{ll} \partial _t \rho _t=\triangle \rho _t-\chi \nabla \cdot (\rho _t\nabla c_t),\quad x\in {\mathbb R}^d, t>0,\\ -\triangle c_t=\rho _t,\\ \rho _0 \quad \text { is given}, \end{array}\right. } \end{aligned}$$

where $\rho _t(x)$ denotes the bacteria density, and $c_t(x)$ represents the chemical substance concentration. The constant $\chi >0$ denotes the chemo-sensitivity or response of the bacteria to the chemical substance. From a mathematical point of view, this equation displays many interesting effects and it has become a topic of intense mathematical research. An important feature of this equation is the competition between the diffusion $\triangle \rho _t$ and the nonlocal aggregation $-\chi \nabla \cdot (\rho _t\nabla c_t)$. Depending on the choice of the initial mass $m_0:=\int _{\mathbb {R}^d}\rho _0(x) \, \mathrm{d} x$ and the chemo-sensitivity $\chi$, the solutions to the KS equation may exist globally or blow-up in finite time. In particular, for sufficiently smooth initial conditions, the existence of solutions was verified by Jäger and Luckhaus (1992): if $m_0\chi$ is large, then solutions are local in time, and they are global in time if $m_0\chi$ is small. For the two-dimensional case, Dolbeault and Perthame (2004) completed the result of Jäger and Luckhaus (1992) by providing an exact value for the critical mass: classical solutions to (1.1) blow-up in finite time when $m_0\chi >8\pi$, and there exists a global in time solution of (1.1) when $m_0\chi <8\pi$. For the case with $m_0\chi =8\pi$, Blanchet et al. (2008) showed that global solutions blow-up in infinite time converging toward a delta diarc distribution at the center of mass. There is an extensive literature on KS systems and their variations, which is out of the scope of this paper. A comprehensive survey on known results related to the KS model from 1970 to 2000 can be found in Horstmann (2003). We also refer to (Hillen and Painter 2009; Perthame 2006; Biler 2018) among many others for more recent developments.

It is also well known that the KS equation (1.1) can be derived from a system of interacting particles $\{(X_t^i)_{t\ge 0}\}_{i= 1}^N$ satisfying the following form of stochastic differential equations (SDEs):

1.2 $$\begin{aligned} \mathrm{d} X_t^i=\frac{\chi }{N-1}\sum _{j\ne i}^{N}F(X_t^i-X_t^j) \, \mathrm{d} t+\sqrt{2}\mathrm{d}B_t^i,\quad i=1,\ldots ,N,\quad t>0, \end{aligned}$$

where the process $(X_t^i)_{t\ge 0}$ denotes the trajectory of the *i*-th particle, the function *F* models the pairwise interaction between particles and $\{(B_t^i)_{t\ge 0}\}_{i= 1}^N$ are *N* independent Wiener processes. The rigorous derivation of the KS equation, for example (1.1), from the microscopic particle system, e.g., (1.2), through the propagation of chaos as $N\rightarrow \infty$ may be found in Huang and Liu (2017a, 2017b), Fournier and Jourdain (2017), Haškovec and Schmeiser (2011), Huang et al. (2019), Fetecau et al. (2019), Bresch et al. (2019). For a review of the topic of the propagation of chaos and the mean-field limit, we refer the readers to Jabin et al. (2017), Carrillo (2014) and the references therein. An asymptotic method, inspired by Hilbert’s sixth problem (Hilbert 1902), can also be applied to derive models at the macro-scale (PDEs) from the underlying description at the micro-scale (particle systems); see Bellomo (2016), Burini and Chouhad (2019) for instance.

However, for the classical deterministic KS equation (1.1), the associated particle system (1.2) is only subject to the idiosyncratic noises that are independent from one particle to another, and the effect of the idiosyncratic noises averages out, leading to the deterministic nature of Eq. (1.1). In addition to such idiosyncratic noises, this paper studies the particle systems allowing for common/environmental noises, and the limiting density function satisfies a stochastic partial differential equation of KS type which is new to the best of our knowledge. Common environmental noises (such as temperature, light and sound) are intrinsic to a more realistic setting such as culturing bacteria .

Let $(\Omega , {\mathcal {F}}, ({\mathcal {F}}_t)_{t\ge 0},\mathbb P)$ be a complete filtered probability space where the $d'$-dimensional Wiener processes $\{(B_t^i)_{t\ge 0}\}_{i=1}^N$ are independent of each other as well as of a $d'$-dimensional Wiener process $(W_t)_{t\ge 0}$.^1^ The initial data $\zeta ^{i}$, $i=1,2,\dots ,N$ are independently and identically distributed (i.i.d.) with a common density function $\rho _0$ and are independent of $\{(B_t^i)_{t\ge 0}\}_{i=1}^{N}$ and $(W_t)_{t\ge 0}$. Denote by $(\mathcal {F}^W_t)_{t\ge 0}$ the augmented filtration generated by $(W_t)_{t\ge 0}$.

As the mean-field limit from the interacting particle system that allows for both idiosyncratic and common noises, the stochastic aggregation–diffusion equation of Keller–Segel (KS) type, also called stochastic KS equation, is of the following form:

1.3 $$\begin{aligned} {\left\{ \begin{array}{ll} \,\mathrm {d}\rho _t =\frac{1}{2}\sum _{i,j=1}^dD_{ij}\left( \rho _t\sum _{k=1}^{d'}(\nu ^{ik}_t\nu ^{jk}_t+ \sigma ^{ik}_t\sigma ^{jk}_t)\right) \, \mathrm{d} t-\chi \nabla \cdot (\nabla c_t\rho _t) \, \mathrm{d} t\\ \quad -\sum _{i=1}^{d}D_i\left( \rho _t \sum _{k=1}^{d'}\sigma ^{ik}_t\,\mathrm {d}W_t^k\right) , \\ -\triangle c_t+c_t=\rho _t,\\ \rho _0 \text { is given}, \end{array}\right. } \end{aligned}$$

where $D_{ij}:=\frac{\partial ^2}{\partial x_i\partial x_j}$, $D_{i}:=\frac{\partial }{\partial x_i}$, and the leading coefficients $\nu$ and $\sigma$ are deterministic functions from $[0,T]\times \mathbb {R}^d$ to $\mathbb {R}^{d\times d'}$. One may solve the second equation for the chemical concentration:

1.4 $$\begin{aligned} c_t=(I-\triangle )^{-1}\rho _t=\mathcal {G}*\rho _t(x), \end{aligned}$$

with $\mathcal {G}$ being the Bessel potential, and it follows that $\nabla c_t=\nabla \mathcal {G} *\rho _t$ where $\nabla \mathcal {G}$ is called the interaction force. The underlying regularized interacting particle system has the form:

1.5 $$\begin{aligned} {\left\{ \begin{array}{ll} \,\mathrm {d}X_t^{i,\varepsilon } = \frac{\chi }{N-1}\sum \limits _{j\ne i}^N\nabla \mathcal {G}_{\varepsilon }(X_t^{i,\varepsilon }-X_t^{j,\varepsilon }) \, \mathrm{d} t+\nu _t(X_t^{i,\varepsilon }) \,\mathrm {d}B_t^i+\sigma _t(X_t^{i,\varepsilon })\,\mathrm {d}W_t, \quad i=1\ldots ,N,\quad t>0,\\ X_0^{i,\varepsilon } =\zeta ^i, \end{array}\right. } \end{aligned}$$

where

$$\begin{aligned} \mathcal {G}_{\varepsilon }(x)=\psi _\varepsilon *\mathcal {G}(x)=\int _{\mathbb {R}^d} \mathcal {G}(y)\psi _\varepsilon (x-y )\,\mathrm{d}y, \quad x\in \mathbb {R}^d,\,\,\varepsilon >0, \end{aligned}$$

is the regularized Bessel potential with the mollifier function $\psi _\varepsilon (x):=\frac{1}{\varepsilon ^d}\psi (\frac{x}{\varepsilon })$ satisfying

1.6 $$\begin{aligned} 0\le \psi \in C_c^\infty ({\mathbb R}^d),\quad \text{ supp } \psi \subseteq B(0,1),\quad \int _{B(0,1)}\psi (x) \, \mathrm{d} x=1. \end{aligned}$$

We mention here relevant work (Cattiaux et al. 2016; Fournier and Jourdain 2017) for the existence of solutions to the non-mollified stochastic particle system (1.2). Especially in Fournier and Jourdain (2017), Proposition 4), they proved that for any $N \ge 2$ and $T>0$, if $\{(X_t^i)_{t\ge 0}\}_{i= 1}^N$ is the solution to (1.2), then

$$\begin{aligned} \mathbb {P}\left( \exists s \in [0, T], \exists 1 \le i<j \le N: X_{s}^{i}=X_{s}^{j}\right) >0, \end{aligned}$$

i.e., the singularity of the drift term is visited and the particle system is not clearly well-defined. Therefore, in order to obtain a global strong solution to the interacting particle system, we regularize the singular force term $\nabla \mathcal {G}$.

In contrast with the classical KS models (1.1) and (1.2), which only allow for the idiosyncratic noise $(B^i_t)_{t\ge 0}$ that is independent from one particle to another, the stochastic systems (1.3) and (1.5) are additionally subject to common noise $(W_t)_{t\ge 0}$, accounting for the common environment where the particles evolve. This common noise leads to the stochastic integrals in stochastic KS equation (1.3), whose (continuous) martingale property and unboundedness result in the inapplicability of classical analysis for deterministic KS equations. In addition, the diffusion coefficients $\sigma$ and $\nu$ are time-state dependent; along the same lines, a general model may allow for diffusion incorporating L$\acute{\text {e}}$vy type noises and/or dependence on the density (for instance, see Burini and Chouhad 2019; Escudero 2006; Huang and Liu 2016 for discussions on deterministic KS models with flux limited or fractional diffusion), although we will not seek such a generality herein.

In this paper, we first prove the existence and uniqueness results for both *weak and strong* solutions to SPDE (1.3). Basically, over a given finite time interval [0, *T*] when the $L^4$-norm of $\rho _0$ is sufficiently small, the weak solution exists uniquely and its regularity may be increased for regular initial value $\rho _0$ (see Theorems 3.2 and 3.3). Then, based on a duality analysis of forward and backward SPDE, we prove that the following stochastic differential equations (SDEs) of McKean–Vlasov type:

1.7 $$\begin{aligned} {\left\{ \begin{array}{ll} \,\mathrm {d}Y^i_t= \chi \nabla \mathcal {G}*\rho ^i_t(Y^i_t) \, \mathrm{d} t+\nu (Y^i_t) \,\mathrm {d}B^i_t+\sigma (Y^i_t)\,\mathrm {d}W_t,\quad i=1,\ldots ,N, \quad t>0,\\ \rho ^i_t \text{ is } \text{ the } \text{ conditional } \text{ density } \text{ of } Y^i_t \text{ given } \mathcal {F}_t^W,\\ Y^i_0=\zeta ^i, \end{array}\right. } \end{aligned}$$

has a unique solution with the conditional density $\rho ^i_t$ of $Y_t^i$ given the common noise $W_t$ *existing and satisfying* SPDE (1.3); see Theorem 4.1. Here by the conditional density $\rho ^i_t$ of $Y^i_t$ given $\mathcal {F}_t^W$, we mean that

$$\begin{aligned} \mathbb {E}[Y^i_t\in \, \mathrm{d} x|\mathcal {F}_t^W]=\rho ^i_t(x) \, \mathrm{d} x, \end{aligned}$$,

i.e., for any $\varphi \in C_b(\mathbb {R}^d)$, it holds that

$$\begin{aligned} \mathbb {E}[\varphi (Y^i_t)|\mathcal {F}_t^W]=\int _{\mathbb {R}^d}\varphi (x)\rho ^i_t(x) \, \mathrm{d} x. \end{aligned}$$

Finally, we prove that the solution $\{(X_t^{i,\varepsilon })_{t\ge 0}\}_{i=1}^N$ of the particle system (1.5) well approximates that of (1.7), which indicates the mean-field limit result, i.e., the empirical measure

$$\begin{aligned} \rho _t^{\varepsilon ,N}:=\frac{1}{N}\sum _{i=1}^{N}\delta _{X_t^{i,\varepsilon }}, \end{aligned}$$

associated with the particle system (1.5) converges weakly to the unique solution $\rho$ to SPDE (1.3) as $N\rightarrow \infty$ and $\varepsilon \rightarrow 0^+$; see Theorem 5.1 and Corollary 2.

In view of SPDE (1.3) and the particle system (1.5), one may see that when the particle number *N* tends to infinity, the effect of the idiosyncratic noises averages out, while the effect of common noises does not, leading to the stochastic nature of the limit distribution characterized by SPDE (1.3). We refer to Bensoussan et al. (2013), Carmona et al. (2016), Carmona and Delarue (2018), Coghi and Flandoli (2016) for different models with common noise in the literature. In particular, in a closely related work (Coghi and Flandoli 2016), the authors study the propagation of chaos for an interacting particle system subject to a common environmental noise but with a uniformly Lipschitz continuous potential, and in Choi and Salem (2019), the stochastic mean-field limit of the Cucker–Smale flocking particle system is obtained for a special class of noises. In contrast to the existing literature concerning common noise, the main difficulties in dealing with the proposed stochastic KS models are from the Bessel potential $\mathcal {G}$ which entails the singularity of the drift of SDE (1.7) and the KS type nonlinear and nonlocal properties of SPDE (1.3); in particular, the KS type nonlinear term $-\chi \nabla \cdot ((\nabla \mathcal {G}*\rho _t) \rho _t)$ prevents us from adopting the existing methods in the SPDE literature. Accordingly, the existence and uniqueness of solution to SPDE (1.3) is established within sufficiently regular spaces under a divergence-free assumption on coefficient $\sigma$, and we prove that the conditional density exists and satisfies equation (1.3) with a new method based on duality analysis. In addition, for the mean-field limit result, we also introduce regularization with a mollifier function in the particle system (1.5). In this paper, the approaches mix and develop the existing probability theory and stochastic analysis, (S)PDE theory, and the duality analysis in nonlinear filtering theory. Given the outstanding interests shown in the mathematical analysis of biological phenomena, we hope this article will set the stage for further studies on stochastic aggregation-diffusion type equations, opening new perspectives and motivating applied mathematicians to expand the research on this class of models to novel applications.

The rest of the paper is organized as follows. In Sect. 2, we set some notations, present some auxiliary results and give the standing assumptions on the diffusion coefficients. Section 3 is then devoted to the proof of the existence and uniqueness of the *weak and strong* solution to stochastic KS equation (1.3) in certain regular spaces. On the basis of the well-posedness of SPDE (1.3), we prove the existence and uniqueness of the strong solution to SDE (1.7) in Sect. 4. Finally, the mean-field limit result is addressed in Sect. 5.

## Preliminaries

### Notations

The set of all the integers is denoted by $\mathbb {Z}$, with $\mathbb {Z}^+$ the subset of the strictly positive elements. Denote by $|\cdot |$ (respectively, $\langle \cdot ,\cdot \rangle$ or $\cdot$) the usual norm (respectively, scalar product) in finite-dimensional Hilbert space such as $\mathbb R,\mathbb R^k,\mathbb R^{k\times l}$, $k,l\in \mathbb {Z}^+$. We use $\Vert f\Vert _p$ for the $L^p$ $(1\le p\le \infty )$ norm of a function *f*.

Define the set of multi-indices

$$\begin{aligned} \mathcal {A}:=\{\alpha =(\alpha _1,\ldots ,\alpha _d): \alpha _1, \ldots , \alpha _d \text { are nonnegative integers}\}. \end{aligned}$$

For any $\alpha \in \mathcal {A}$ and $x=(x_1,\ldots ,x_d)\in \mathbb {R}^d,$ denote

$$\begin{aligned} |\alpha |=\sum _{i=1}^d \alpha _i,\ x^{\alpha }:=x_1^{\alpha _1}x_2^{\alpha _2}\ldots x_d^{\alpha _d},\ D^{\alpha }:=\frac{\partial ^{|\alpha |}}{\partial x_1^{\alpha _1}\partial x_2^{\alpha _2}\ldots \partial x_d^{\alpha _d}}. \end{aligned}$$

For each Banach space $(\mathcal {X},\Vert \cdot \Vert _{\mathcal {X}})$, real $q\in [1,\infty ]$, and $0\le t <\tau \le T$, we denote by $S_{\mathcal F}^q([t,\tau ];\mathcal {X})$ the set of $\mathcal {X}$-valued, $\mathcal {F}_{t \in [0,T]}$-adapted and continuous processes $\{X_s\}_{s\in [t,\tau ]}$ such that

$$\begin{aligned} \Vert X\Vert _{S_{\mathcal F}^q([t,\tau ];\mathcal {X})}:= \left\{ \begin{array}{l} \left( \mathbb {E}\Big [ \sup _{s\in [t,\tau ]} \Vert X_s\Vert _{\mathcal {X}}^q \Big ]\right) ^{1/q},\quad q\in [1,\infty );\\ {{\,\mathrm{ess\,sup}\,}}_{\omega \in \Omega } \sup _{s\in [t,\tau ]} \Vert X_s\Vert _{\mathcal {X}},\quad q=\infty . \end{array}\right. \end{aligned}$$

$L_{\mathcal F}^q(t,\tau ;\mathcal {X})$ denotes the set of (equivalent classes of) $\mathcal {X}$-valued predictable processes $\{X_s\}_{s\in [t,\tau ]}$ such that

$$\begin{aligned} \Vert X\Vert _{L^q_{\mathcal F}(t,\tau ;\mathcal {X})}:= \left\{ \begin{array}{l} \left( \mathbb {E}\Big [ \int _t^\tau \Vert X_s\Vert _{\mathcal {X}}^q\,\mathrm{d}s \Big ]\right) ^{1/q},\quad q\in [1,\infty );\\ {{\,\mathrm{ess\,sup}\,}}_{(\omega ,s)\in \Omega \times {[t,\tau ]}} \Vert X_s\Vert _{\mathcal {X}},\quad q=\infty . \end{array}\right. \end{aligned}$$

Both $\left( S_{\mathcal F}^q([t,\tau ];\mathcal {X}),\Vert \cdot \Vert _{S^q([t,\tau ]\mathcal {X})}\right)$ and $\left( L_{\mathcal F}^q(t,\tau ;\mathcal {X}),\Vert \cdot \Vert _{L_{\mathcal F}^q(t,\tau ;\mathcal {X})}\right)$ are Banach spaces, and they are well defined with the filtration $(\mathcal {F}_t)_{t\ge 0}$ replaced by $(\mathcal {F}_t^W)_{t\ge 0}$.

### Auxiliary Results and Assumptions

We first recall some properties of the Bessel potential introduced in (1.4). For $p\in [1,\infty ]$, denote by $L^p=L^p(\mathbb {R}^d)$ the usual Lebesgue integrable spaces with norm $\Vert \cdot \Vert _p$. Then, for $p\in (1,\infty )$ and $m\in \mathbb {R}$, we may define the space of Bessel potentials (or the Sobolev space with fractional derivatives) (Triebel 1983, p. 37) as

$$\begin{aligned} \mathcal {H}_p^m(\mathbb {R}^d)=\left\{ f\big |~\Vert f\Vert _{\mathcal {H}_p^m}:=\Vert \mathscr {F}^{-1}[(1+|\omega |^2)^{\frac{m}{2}}\mathscr {F}(f)]\Vert _p<\infty \right\} , \end{aligned}$$

where $\mathscr {F}$ is the Fourier transformation. Namely, $\mathcal {H}_p^m(\mathbb {R}^d)$ (simply written as $\mathcal {H}_p^m$) is defined as space of functions *f* such that $(1-\triangle )^{\frac{m}{2}}f\in L^p(\mathbb {R}^d)$. In (1.4), if $\rho _t\in L^p$ with $1<p<\infty$, then $c_t \in \mathcal {H}_p^2$. In addition, it holds that

$$\begin{aligned} \left\Vert c_t \, \right\Vert _{\mathcal {H}_p^2}=\left\Vert \mathscr {F}^{-1}\left[ (1+|\omega |^2)\mathscr {F}[c_t]\right] \, \right\Vert _{p}=\left\Vert \rho _t \, \right\Vert _{p}. \end{aligned}$$

Due to the equivalence between the Bessel potential space $\mathcal {H}_p^k(\mathbb {R}^d)$ and the Sobolev space $W^{k,p}(\mathbb {R}^d)$ $(k\in \mathbb {N})$, we have

2.1 $$\begin{aligned} \left\Vert \mathcal {G}*\rho _t \, \right\Vert _{W^{2,p}}=\left\Vert c_t \, \right\Vert _{W^{2,p}}\le C\left\Vert \rho _t \, \right\Vert _{p}. \end{aligned}$$

Here, the Sobolev space $W^{{k,p}}(\mathbb {R}^d )$ is defined as

$$\begin{aligned} W^{k,p}(\mathbb {R}^d )=\left\{ u\in L^{p}(\mathbb {R}^d )\big |~D^{\alpha }u\in L^{p}(\mathbb {R}^d ),\,\,\forall ~ |\alpha |\le k\right\} \end{aligned}$$

and

$$\begin{aligned} \Vert u\Vert _{W^{k, p}}:=\left\{ \begin{array}{ll}\left( \sum _{|\alpha | \leqslant k}\left\| D^{\alpha } u\right\| _{L^{p}(\mathbb {R}^d)}^{p}\right) ^{\frac{1}{p}} &{} 1 \leqslant p<\infty , \\ \max _{|\alpha | \leqslant k}\left\| D^{\alpha } u\right\| _{L^{\infty }(\mathbb {R}^d)} &{} p=\infty . \end{array}\right. \end{aligned}$$

On the other side, notice that

$$\begin{aligned} (I-\triangle )^{-1}=(-\triangle )^{-1}-(-\triangle )^{-1}(I-\triangle )^{-1}. \end{aligned}$$

Thus, we may split the Bessel potential into the Newtonian potential $\varPhi$ and a function $\varPsi$ such that $\mathscr {F}(\varPsi )(\omega )=-\frac{1}{\omega ^2(1+\omega ^2)}$, which implies that $\varPsi \in L^\infty (\mathbb {R}^d)$ $(d=3)$ or $\nabla \varPsi \in L^\infty (\mathbb {R}^d)$ $(d=2)$. Namely, one has

2.2 $$\begin{aligned} \mathcal {G}(x)=\varPhi (x)+\varPsi (x), \end{aligned}$$

where

$$\begin{aligned} \varPhi (x)=\left\{ \begin{array}{ll}{\frac{C_{d}}{|x|^{d-2}},} &{} { \text{ if } d \ge 3} \\ {-\frac{1}{2 \pi } \ln |x|,} &{} { \text{ if } d=2}\end{array}\right. \end{aligned}$$

is the Newtonian potential. It then follows that for any $\alpha \in \mathcal A$ with $|\alpha |\ge 1$, there holds

2.3 $$\begin{aligned} \left\Vert D^\alpha (\nabla \mathcal {G}_\varepsilon ) \, \right\Vert _\infty&\le C_{\alpha }\varepsilon ^{1-d-|\alpha |}+ {\left\{ \begin{array}{ll} C_{\alpha ,\left\Vert \varPsi \, \right\Vert _\infty }\varepsilon ^{-1-|\alpha |},\quad &{}\text{ when } d=3\,;\\ C_{\alpha ,\left\Vert \nabla \varPsi \, \right\Vert _\infty }\varepsilon ^{-|\alpha |},\quad &{}\text{ when } d=2 \end{array}\right. } \nonumber \\&\le C_\alpha \varepsilon ^{1-d-|\alpha |}. \end{aligned}$$

Here, we have used the estimate $\left\Vert D^\alpha (\nabla \varPhi _\varepsilon ) \, \right\Vert _\infty \le C_{\alpha }\varepsilon ^{1-d-|\alpha |}$ from Huang and Liu (2017b, Lemma 2.1).

Following are the standing assumptions on the coefficients $\nu$ and $\sigma$.

#### Assumption 1

Given $T>0$ any arbitrary time horizon and $d=2,3$ , the measurable diffusion coefficients $\sigma ,\,\nu : [0,T]\times \mathbb {R}^d \longrightarrow \mathbb {R}^{d\times d'}$ satisfy

(i) There exists a positive constant $\lambda$ such that
  $$\begin{aligned} \sum _{i,j=1}^d \sum _{k=1}^{d'}\nu ^{ik}_t(x)\nu ^{jk}_t(x)\xi ^i\xi ^j\ge \lambda |\xi |^2 \end{aligned}$$
  holds for all $x,\xi \in \mathbb R^d$ and all $t\ge 0$;
(ii) There exist $m\in \mathbb Z^+$ and real $\varLambda >0$ such that for all $t\in [0,T]$ there holds
  $$\begin{aligned}&\nu ^{ik}_t(\cdot ),\,\sigma ^{ik}_t(\cdot )\in C^m , \,\,\text {for } i=1,\dots ,d, \,k=1,\dots ,d',\\&\quad \text {and}\quad \sum _{i=1}^d\sum _{k=1}^{d'} \Vert \sigma ^{ik}_t(\cdot )\Vert _{C^m}+\Vert \nu ^{ik}_t(\cdot )\Vert _{C^m} \le \varLambda , \end{aligned}$$
  where the $C^m$ norm is defined as $\Vert f\Vert _{C^m}=\sum _{|\alpha |\le m}\Vert D^\alpha f\Vert _\infty$.
(iii) For all $(t,x)\in [0,T]\times \mathbb R^d$ and $k=1,2,\dots ,d'$,
  $$\begin{aligned} \sum _{i=1}^dD_i\sigma ^{ik}_t(x)=0. \end{aligned}$$

#### Remark 2.1

The assumption (i) ensures the superparabolicity of the concerned SPDE, and the boundedness and regularity requirements in (ii) are placed for unique existence of certain regular solutions of SPDE. The readers are referred to Krylov (1999) for more discussions. The divergence-free condition (iii) may be thought of as a technical one for the well-posedness of SPDE (1.3) (see Remark 3.1); on the other hand, the common noise in the stochastic integral term $\sigma _t(X_t^{i,\varepsilon })\,\mathrm{d}W_t$ induces the fluctuations of the velocity field (of the *i*th particle) formally written as $v^{i}_t=\sigma _t(X_t^{i,\varepsilon })\frac{\mathrm{d}W_t}{\mathrm{d}t}$ and in this way, the divergence-free condition means that such fluctuations are of incompressible type. In fact, such kind of divergence-free conditions have been existing in the literature; refer to Brzezniak et al. (2016), Coghi and Flandoli (2016) for more clear and elegant arguments.

In the remaining part of the work, we shall use *C* to denote a generic constant whose value may vary from line to line, and when needed, a bracket will follow immediately after *C* to indicate what parameters *C* depend on. By $A\hookrightarrow B$, we mean that normed space $(A,\Vert \cdot \Vert _{A})$ is embedded into $(B,\Vert \cdot \Vert _B)$ with a constant *C* such that

$$\begin{aligned} \Vert f\Vert _B\le C \Vert f\Vert _A,\,\,\,\forall f\in A. \end{aligned}$$

For readers’ convenience, we list Sobolev’s embedding theorem in the following lemma, see, e.g., Triebel (1983, p. 129, p. 131) and Brezis (2010, Chapter 9).

#### Lemma 2.1

There holds the following assertions:

(i) For integer $n>d/q+k$ with $k\in \mathbb {N}$ and $q\in (1,\infty )$, we have $W^{n,q}(\mathbb {R}^d)\hookrightarrow C^{k,\delta }(\mathbb {R}^d)$, for any $\delta \in (0,(n-d/q-k)\wedge 1).$
(ii) If $1<p_0<p_1<\infty$ and $-\infty<s_1<s_0<+\infty$ such that $\frac{d}{p_0}-s_0=\frac{d}{p_1}-s_1$, then $\mathcal {H}_{p_0}^{s_0}(\mathbb {R}^d)\hookrightarrow \mathcal {H}_{p_1}^{s_1}(\mathbb {R}^d)$ (with Sobolev spaces as special cases ).

## Existence and Uniqueness of the Solution to SPDE (1.3)

This section is devoted to the global existence and uniqueness of the solution to nonlinear SPDE (1.7).

As already noted in (2.1), if $\rho _t\in L^4$, then it holds that

3.1 $$\begin{aligned} \left\Vert c_t \, \right\Vert _{W^{2,4}}=\left\Vert \mathcal {G}*\rho _t \, \right\Vert _{W^{2,4}}\le S_d\left\Vert \rho _t \, \right\Vert _{4}. \end{aligned}$$

A direct result of Sobolev’s embedding theorem implies

3.2 $$\begin{aligned} \left\Vert c_t \, \right\Vert _{W^{1,\infty }}=\left\Vert \mathcal {G}*\rho _t \, \right\Vert _{W^{1,\infty }}\le \left\Vert \mathcal {G}*\rho _t \, \right\Vert _{W^{2,4}}\le S_d\left\Vert \rho _t \, \right\Vert _{4}, \end{aligned}$$

where $S_d$ depends only on *d*.

Before stating the theorem about the well-posedness, we introduce the definition of solutions to SPDE (1.3). Denote by $C_c^2(\mathbb {R}^d)$ the space of compactly supported functions having up to second-order continuous derivatives.

### Definition 3.1

A family of random functions $\{\rho _t(\omega ):~t\ge 0,\omega \in \Omega \}$ lying in $S_{\mathcal {F}^W}^{\infty }([0,T];L^1\cap L^4(\mathbb {R}^d))$ is a solution to Eq. (1.3) if $\rho _t$ satisfies the following stochastic integral equation for all $\varphi \in C_c^2(\mathbb {R}^d)$,

3.3 $$\begin{aligned} \langle \rho _t,\varphi \rangle&=\langle \rho _0,\varphi \rangle +\chi \int _0^t\langle \rho _s,\nabla \varphi \cdot \nabla c_s\rangle \, \mathrm{d} s+\int _0^t\left\langle \rho _s,\sum _{i=1}^{d}D_i \varphi \sum _{k=1}^{d'}\sigma _s^{ik}\,\mathrm {d}W_s^k\right\rangle \nonumber \\&\quad +\frac{1}{2}\int _0^t\left\langle \rho _s,\sum _{i,j=1}^dD_{ij} \varphi \sum _{k=1}^{d'}(\nu _s^{ik}\nu _s^{jk}+ \sigma _s^{ik}\sigma _s^{jk})\right\rangle \, \mathrm{d} s. \end{aligned}$$

### Theorem 3.2

Let Assumption 1 hold with $m=2$. Assume $0\le \rho _0\in L^1\cap \mathcal {H}^{\frac{1}{2}}_{4}(\mathbb {R}^d)$^2^ with $\left\Vert \rho _0 \, \right\Vert _1=1$. For each $T>0$, there exists a $\kappa >0$ depending only on $T,\chi ,\lambda ,\varLambda$ and *d* such that if $\Vert \rho _0\Vert _{4}\le \kappa$, SPDE (1.3) admits a unique nonnegative solution in

3.4 $$\begin{aligned} {\mathbb {M}}:= & {} L^2_{\mathcal F^W} (0,T;W^{1,2}(\mathbb {R}^d))\cap L^4_{\mathcal F^W} (0,T;W^{1,4}(\mathbb {R}^d)) \nonumber \\&\cap S_{\mathcal F^W}^{\infty }([0,T];L^1\cap L^4(\mathbb {R}^d)). \end{aligned}$$

### Proof

The proof is based on delicate estimates of the solution and the latest developments of $L^p$-theory of SPDE. First, let

$$\begin{aligned} \mathbb B:=\left\{ u\in S_{\mathcal F^W}^{\infty } ([0,T];L^4(\mathbb {R}^d)): \, \Vert u\Vert _{S_{\mathcal F^W}^{\infty } ([0,T];L^4(\mathbb {R}^d))} \le \ell \kappa \right\} , \end{aligned}$$

with metric $d(u,v)=\left\Vert u-v \, \right\Vert _{S_{\mathcal F^W}^{\infty } ([0,T]; L^4(\mathbb {R}^d))}$, and the positive constants $\kappa$ and $\ell$ are to be determined.

Suppose $\Vert \rho _0\Vert _{4}\le \kappa$. Now, we define a map $\mathcal {T}: \mathbb {B}\rightarrow S_{\mathcal F^W}^{\infty } ([0,T];L^4(\mathbb {R}^d))$ as follows: For each $\xi \in \mathbb {B}$, let $\mathcal {T}(\xi ):=\rho ^\xi$ be the solution to the following linear SPDE:

3.5 $$\begin{aligned} \left\{ \begin{array}{l} \,\mathrm {d}\rho _t =\left[ \frac{1}{2}\sum _{i,j=1}^dD_{ij}(\rho _t\sum _{k=1}^{d'}(\nu _t^{ik}\nu _t^{jk}+ \sigma _t^{ik}\sigma _t^{jk})) -\chi \nabla \cdot ((\nabla \mathcal {G}*\xi _t) \rho _t) \right] \, \mathrm{d} t\\ \quad -\sum _{i=1}^dD_i( \rho _t \sum _{k=1}^{d'}\sigma _t^{ik}\,\mathrm {d}W_t^k),\\ \rho _0\text { is given}. \end{array}\right. \end{aligned}$$

Indeed, as Assumption 1 holds with $m=2$, one may write SPDE (3.5) as a non-divergence form:

3.6 $$\begin{aligned} \left\{ \begin{array}{l} \,\mathrm {d}\rho _t =\left[ \frac{1}{2}\sum _{i,j=1}^d\sum _{k=1}^{d'}(\nu _t^{ik}\nu _t^{jk}+ \sigma _t^{ik}\sigma _t^{jk})D_{ij}\rho _t \, \mathrm{d} t+F_t(\rho _t) \right] \, \mathrm{d} t\\ \quad -\sum _{i=1}^d \sum _{k=1}^{d'} \sigma _t^{ik} D_i \rho _t\,\mathrm {d}W_t^k,\\ \rho _0\text { is given}, \end{array}\right. \end{aligned}$$

with

$$\begin{aligned} F_t(\rho _t):&=\frac{1}{2}\sum _{i,j=1}^d D_{i}\left( \rho _t\sum _{k=1}^{d'}D_j(\nu _t^{ik}\nu _t^{jk}+ \sigma _t^{ik}\sigma _t^{jk})\right) \\&\quad +\frac{1}{2}\sum _{i,j=1}^d D_j\rho _t\sum _{k=1}^{d'}D_i(\nu _t^{ik}\nu _t^{jk}+ \sigma _t^{ik}\sigma _t^{jk}) \\&\quad -\chi \nabla \cdot ((\nabla \mathcal {G}*\xi _t) \rho _t), \end{aligned}$$

where we have used Assumption 1 (iii) for the stochastic integral, i.e.,

3.7 $$\begin{aligned} \sum _{j=1}^dD_j(\sigma _s^{jk} \rho _s)=\sum _{j=1}^d\rho _s D_j\sigma _s^{jk} +\sum _{j=1}^dD_j\rho _s\sigma _s^{jk} =\sum _{j=1}^dD_j\rho _s \sigma _s^{jk}. \end{aligned}$$

For each $\xi \in \mathbb B$ and $\rho _t\in L^p(\mathbb {R}^d)$ with $p\in \{2,4\}$, relation (3.2) indicates that

$$\begin{aligned} \Vert (\nabla \mathcal {G}*\xi _t)\rho _t\Vert _{p}&\le \Vert \nabla \mathcal {G}*\xi _t\Vert _{{\infty }} \Vert \rho _t\Vert _{p} \le S_d\left\Vert \xi _t \, \right\Vert _4\Vert \rho _t\Vert _{p}\le \ell S_d\kappa \Vert \rho _t\Vert _{p}, \quad \text {a.s., for all }t\in [0,T]. \end{aligned}$$

This together with Assumption 1 allows us, through standard computations, to check that the conditions of the $L^p$-theory of SPDE (see Krylov 1999, Theorems 5.1 and 7.1 for the case when $n=-1$ therein) and the maximum principle (Krylov 1999, Theorem 5.12) are satisfied and we conclude that the linear SPDE (3.5) admits a unique solution $\rho ^{\xi }$ which is nonnegative and lying in $L^p_{\mathcal F^W} (0,T;W^{1,p}(\mathbb {R}^d)) \cap S^p_{\mathcal F^W} ([0,T];L^p(\mathbb {R}^d))$, $p\in \{2,4\}$.

Next, we check that $\rho ^{\xi }\in S^\infty _{\mathcal F^W}([0,T];L^1\cap L^4(\mathbb {R}^d))$ and without causing confusion we drop the superscript $\xi$. It is easy to see that the solution of (3.5) has the property of conservation of mass, i.e.,

$$\begin{aligned} \left\Vert \rho _t \, \right\Vert _1=\left\Vert \rho _0 \, \right\Vert _1=1\quad \text {a.s.}. \end{aligned}$$

Applying the Itô formula for $L^p$-norms in Krylov (2010, Theorem 2.1), we have for any $0<t\le T$

3.8 $$\begin{aligned}&\Vert \rho _{t}\Vert _{4}^4-\Vert \rho _0\Vert _{4}^4 \nonumber \\&\quad =\int _0^{t } \bigg (\sum _{i,j=1}^d\sum _{k=1}^{d'}-\left\langle 6 |\rho _s|^2 D_i\rho _s,\, D_j\left( (\nu _s^{ik}\nu _s^{jk}+\sigma _s^{ik}\sigma _s^{jk})\rho _s\right) \right\rangle \nonumber \\&\quad + 6\sum _{k=1}^{d'}\left\langle |\rho _s|^2 ,\, |\sum _{j=1}^dD_j(\rho _s\sigma _s^{jk} )|^2\right\rangle \nonumber \\&\qquad +12\chi \left\langle \rho _s(\nabla \rho _s),( \nabla \mathcal {G}*\xi _s) \rho _s ^2 \right\rangle \bigg )\,\mathrm{d}s +12\sum _{i=1}^d\sum _{k=1}^{d'}\int _0^{t} \left\langle |\rho _s|^2 D_i\rho _s,\,\sigma _s^{ik}\rho _s \right\rangle \,\mathrm{d}W^k_s \quad \text {a.s.}. \end{aligned}$$

Due to (iii) in Assumption 1, we know that for $k=1,2,\dots ,d'$,

$$\begin{aligned} 12 \sum _{i=1}^d\left\langle |\rho _s|^2 D_i\rho _s,\,\sigma ^{ik}\rho _s \right\rangle = 3\sum _{i=1}^d\left\langle D_i\left( |\rho _s|^4\right) ,\,\sigma ^{ik}_s \right\rangle =-3\left\langle |\rho _s|^4,\,\sum _{i=1}^dD_i\sigma ^{ik}_s \right\rangle =0. \end{aligned}$$

Thus, one has

$$\begin{aligned}&\Vert \rho _{t}\Vert _{4}^4-\Vert \rho _0\Vert _{4}^4 \\&\quad =-\int _0^{t} \sum _{i,j=1}^d\sum _{k=1}^{d'} \left\langle 6 |\rho _s|^2 D_i\rho _s,\, (\nu ^{ik}_s\nu _s^{jk}+\sigma _s^{ik}\sigma _s^{jk})D_j\rho _s \right\rangle \,\mathrm{d}s\\&\qquad -\sum _{i,j=1}^d\sum _{k=1}^{d'}\int _0^{t}\left\langle 6 |\rho _s|^2 D_i\rho _s,\, D_j(\nu ^{ik}_s\nu _s^{jk}+\sigma _s^{ik}\sigma _s^{jk})\rho _s \right\rangle \,\mathrm{d}s \\&\qquad +\int _0^{t} 6 \sum _{k=1}^{d'} \left\langle |\rho _s|^2 ,\, |\sum _{j=1}^dD_j(\rho _s\sigma _s^{jk} ) |^2\right\rangle +12\chi \left\langle \rho _s(\nabla \rho _s),( \nabla \mathcal {G}*\xi _s) \rho _s ^2 \right\rangle \,\mathrm{d}s \quad \text {a.s.}. \end{aligned}$$

Using (iii) in Assumption 1 as in (3.7) again yields that

$$\begin{aligned}&-\sum _{i,j=1}^d\sum _{k=1}^{d'} \left\langle 6 |\rho _s|^2 D_i\rho _s,\, (\nu ^{ik}_s\nu _s^{jk}+\sigma _s^{ik}\sigma _s^{jk})D_j\rho _s \right\rangle \\&\quad =-\sum _{i,j=1}^d\sum _{k=1}^{d'} \left\langle 6 |\rho _s|^2 D_i\rho _s,\, (\nu ^{ik}_s\nu _s^{jk})D_j\rho _s \right\rangle -\sum _{k=1}^{d'} \left\langle 6 |\rho _s|^2 ,\, \left| \sum _{j=1}^dD_j\rho _s \sigma _s^{jk}\right| ^2\right\rangle \\&\quad =-\sum _{i,j=1}^d\sum _{k=1}^{d'} \left\langle 6 |\rho _s|^2 D_i\rho _s,\, (\nu ^{ik}_s\nu _s^{jk})D_j\rho _s \right\rangle -\sum _{k=1}^{d'} \left\langle 6 |\rho _s|^2 ,\,\left| \sum _{j=1}^dD_j(\rho _s\sigma _s^{jk} ) \right| ^2\right\rangle . \end{aligned}$$

Therefore, it holds that

3.9 $$\begin{aligned}&\Vert \rho _{t}\Vert _{4}^4-\Vert \rho _0\Vert _{4}^4 \nonumber \\&\quad =-\int _0^{t} \sum _{i,j=1}^d\sum _{k=1}^{d'} \left\langle 6 |\rho _s|^2 D_i\rho _s,\, (\nu ^{ik}_s\nu _s^{jk})D_j\rho _s \right\rangle \,\mathrm{d}s\nonumber \\&\qquad -\int _0^{t}\sum _{i,j=1}^d\sum _{k=1}^{d'} \left\langle 6 |\rho _s|^2 D_i\rho _s,\, D_j(\nu ^{ik}_s\nu _s^{jk}+\sigma _s^{ik}\sigma _s^{jk})\rho _s \right\rangle \,\mathrm{d}s \nonumber \\&\qquad +12 \chi \int _0^{t} \left\langle \rho _s(\nabla \rho _s),( \nabla \mathcal {G}*\xi _s) \rho _s ^2 \right\rangle \,\mathrm{d}s, \quad \text {a.s.}. \end{aligned}$$

It follows from (i) in Assumption 1 that

$$\begin{aligned} -\sum _{i,j=1}^d\sum _{k=1}^{d'} \left\langle 6 |\rho _s|^2 D_i\rho _s,\, (\nu ^{ik}_s\nu _s^{jk})D_j\rho _s \right\rangle \le -6\lambda \Vert \rho _s\nabla \rho _s\Vert _{2}^2, \end{aligned}$$

and by (ii) in Assumption 1 one has

3.10 $$\begin{aligned}&-6\sum _{i,j=1}^d\sum _{k=1}^{d'} \left\langle |\rho _s|^2 D_i\rho _s,\, D_j(\nu ^{ik}_s\nu _s^{jk}+\sigma _s^{ik}\sigma _s^{jk})\rho _s \right\rangle \nonumber \\&\quad \le 24\varLambda ^2 \left\langle |\rho _s|^2 |\nabla \rho _s|,\, \rho _s \right\rangle \nonumber \\&\quad \le 2\lambda \Vert \rho _s\nabla \rho _s\Vert _{2}^2+\frac{(12\varLambda ^2 )^2}{2\lambda }\Vert \rho _s\Vert _{4}^4 . \end{aligned}$$

We also notice that

3.11 $$\begin{aligned} 12\chi \left\langle \rho _s(\nabla \rho _s),( \nabla \mathcal {G}*\xi _s) \rho _s ^2 \right\rangle&\le 12\chi \Vert \rho _s\nabla \rho _s\Vert _{2} \Vert \rho _s\Vert _{4}^2 \Vert \nabla \mathcal {G}*\xi _s\Vert _{{\infty }} \nonumber \\ (\text {by relation} (3.2))&\le 12\chi S_d \Vert \rho _s\nabla \rho _s\Vert _{2} \Vert \rho _s\Vert _{4}^{2}\left\Vert \xi _s \, \right\Vert _4\le 12\ell \chi S_d\kappa \Vert \rho _s\nabla \rho _s\Vert _{2} \Vert \rho _s\Vert _{4}^{2} \nonumber \\ \text {(by Young's inequality)}&\le 2\lambda \Vert \rho _s\nabla \rho _s\Vert _{2}^2 + \frac{(6\ell \chi S_d\kappa )^2}{2\lambda }\Vert \rho _s\Vert _{4}^{4}. \end{aligned}$$

Collecting above estimates, (3.9) yields that

3.12 $$\begin{aligned}&\Vert \rho _{t}\Vert _{4}^4-\Vert \rho _0\Vert _{4}^4\nonumber \\&\quad \le -6\lambda \int _0^{t} \Vert \rho _s\nabla \rho _s\Vert _{2}^2\,\mathrm{d}s +4\lambda \int _0^{t}\Vert \rho _s\nabla \rho _s\Vert _{2}^2\,\mathrm{d}s + \left( \frac{(12\varLambda ^2)^2}{2\lambda }+\frac{(6\ell \chi S_d\kappa )^2}{2\lambda }\right) \int _0^{t} \Vert \rho _s\Vert _{4}^{4} \,\mathrm{d}s \nonumber \\&\quad \le \left( \frac{(12\varLambda ^2)^2}{2\lambda }+\frac{(6\ell \chi S_d\kappa )^2}{\lambda }\right) \int _0^{t} \Vert \rho _s\Vert _{4}^{4} \,\mathrm{d}s. \end{aligned}$$

Take a sufficiently large $\ell >1$ and relatively small $\kappa _0$^3^ such that whenever $\kappa \le \kappa _0$ it holds that

3.13 $$\begin{aligned} \exp \left\{ \frac{T}{4} \left( \frac{(12\varLambda ^2)^2}{2\lambda }+\frac{(6\ell \chi S_d\kappa )^2}{\lambda }\right) \right\} \le \ell . \end{aligned}$$

Applying Gronwall’s inequality to (3.12) yields that

$$\begin{aligned} \sup _{t\in [0,T]} \Vert \rho _{t}\Vert _{4}&\le \Vert \rho _0\Vert _{4} \exp \left\{ \frac{T}{4} \left( \frac{(12\varLambda ^2)^2}{2\lambda }+\frac{(6\ell \chi S_d\kappa )^2}{\lambda }\right) \right\} ,\\&\le \ell \kappa , \end{aligned}$$

which gives that $\rho \in \mathbb B$.

Fix the constants $\ell$ and $\kappa _0$ as selected above. Let $\kappa \le \kappa _0$. For all $\xi \in \mathbb {B}$, let $\rho ^{\xi }$ be the unique solution of the linear SPDE (3.5). From the discussion above, we get the solution map

$$\begin{aligned} \mathcal T:\quad \mathbb B \rightarrow \mathbb B, \quad \xi \mapsto \rho ^{\xi }. \end{aligned}$$

Next, we show that the map $\mathcal {T}$ is a contraction.

For any $\bar{\xi },\, \hat{\xi }\in \mathbb B$, set $\delta \rho =\rho ^{\bar{\xi }}-\rho ^{\hat{\xi }}$ and $\delta \xi =\bar{\xi }-{\hat{\xi }}$. As before, we apply Itô formula for the $L^4$-norm of $\delta \rho$:

3.14 $$\begin{aligned}&\Vert \delta \rho _{t}\Vert _{4}^4 \nonumber \\&\quad = \int _0^{t}\left( -\sum _{i,j=1}^d\sum _{k=1}^{d'}\left\langle 6 |\delta \rho _s|^2 D_i\delta \rho _s,\, D_j\left( (\nu _s^{ik}\nu _s^{jk}+\sigma _s^{ik}\sigma _s^{jk})\delta \rho _s\right) \right\rangle \right. \nonumber \\&\left. \qquad + 6\sum _{k=1}^{d'}\left\langle |\delta \rho _s|^2 ,\, \left| \sum _jD_j(\delta \rho _s\sigma _s^{jk} )\right| ^2\right\rangle \right) \,\mathrm{d}s \nonumber \\&\qquad +\int _0^t12\chi \left\langle |\delta \rho _s|^2 \nabla \delta \rho _s, \nabla \mathcal {G}*\bar{\xi }_s \rho ^{\bar{\xi }}_s- \nabla \mathcal {G}*{\hat{\xi _s}} \rho ^{\hat{\xi }}_s \right\rangle \,\mathrm{d}s \nonumber \\&\qquad + 12\sum _{i=1}^d\sum _{k=1}^{d'}\int _0^{t} \left\langle |\delta \rho _s|^2 D_i\delta \rho _s,\,\sigma _s^{ik}\delta \rho _s \right\rangle \,\mathrm{d}W^k_s \nonumber \\&\quad = \int _0^{t}\left( -\sum _{i,j=1}^d\sum _{k=1}^{d'} \left\langle 6 |\delta \rho _s|^2 D_i\delta \rho _s,\, (\nu _s^{ik}\nu _s^{jk})D_j\delta \rho _s\right\rangle \right. \nonumber \\&\left. \qquad -\sum _{i,j=1}^d\sum _{k=1}^{d'} \left\langle 6 |\delta \rho _s|^2 D_i\delta \rho _s,\, D_j(\nu ^{ik}_s\nu _s^{jk}+\sigma _s^{ik}\sigma _s^{jk})\delta \rho _s \right\rangle \right) \,\mathrm{d}s \nonumber \\&\qquad + \int _0^t12\chi \left\langle |\delta \rho _s|^2 \nabla \delta \rho _s, \nabla \mathcal {G}*\bar{\xi }_s \rho ^{\bar{\xi }}_s- \nabla \mathcal {G}*\hat{\xi }_s \rho ^{\hat{\xi }}_s \right\rangle \,\mathrm{d}s \nonumber \\&\quad \le -6\lambda \int _0^{t} \Vert \delta \rho _s\nabla \delta \rho _s\Vert _2^2\,\mathrm{d}s +2\lambda \int _0^t\Vert \delta \rho _s\nabla \delta \rho _s\Vert _{2}^2\,ds+\frac{(12\varLambda ^2 )^2}{2\lambda }\int _0^t\Vert \delta \rho _s\Vert _{4}^4\,\mathrm{d}s \nonumber \\&\qquad +\int _0^t12\chi \left\langle |\delta \rho _s|^2 \nabla \delta \rho _s, \nabla \mathcal {G}*\bar{\xi }_s \rho ^{\bar{\xi }}_s- \nabla \mathcal {G}*\hat{\xi }_s \rho ^{\hat{\xi }}_s \right\rangle \,\mathrm{d}s,\quad \text {a.s.}. \end{aligned}$$

Let us compute that

$$\begin{aligned}&12\chi \left\langle |\delta \rho _s|^2 \nabla \delta \rho _s, \nabla \mathcal {G}*\delta \xi _s \rho ^{\bar{\xi }}_s \right\rangle \\&\quad \le 12\chi \Vert \delta \rho _s \nabla \delta \rho _s\Vert _{2} \Vert \delta \rho _s\rho ^{\bar{\xi }}_s\Vert _{2} \Vert \nabla \mathcal {G}*\delta \xi _s \Vert _{{\infty }}\\&\quad \le 12 \chi S_d\Vert \delta \rho _s \nabla \delta \rho _s\Vert _{2} \Vert \delta \rho _s\Vert _{4}\Vert \rho ^{\bar{\xi }}_s\Vert _{4}\left\Vert \delta \xi _s \, \right\Vert _4 \\&\quad \le 2\lambda \Vert \delta \rho _s \nabla \delta \rho _s\Vert _{2}^2+\frac{(6\chi S_d)^2}{2\lambda }\Vert \delta \rho _s\Vert _{4}^2\Vert \rho ^{\bar{\xi }}_s\Vert _{4}^2\left\Vert \delta \xi _s \, \right\Vert _4^2 \\&\quad \le 2\lambda \Vert \delta \rho _s \nabla \delta \rho _s\Vert _{2}^2+\frac{(6\chi S_d)^2}{4\lambda } \left( (\ell \kappa )^{2}\Vert \delta \rho _s\Vert _{4}^4+(\ell \kappa )^{-2}\Vert \rho ^{\bar{\xi }}_s\Vert _{4}^4\left\Vert \delta \xi _s \, \right\Vert _4^4\right) \\&\quad \le 2\lambda \Vert \delta \rho _s \nabla \delta \rho _s\Vert _{2}^2+\frac{(6\ell \chi S_d\kappa )^2}{4\lambda }\Vert \delta \rho _s\Vert _{4}^4+\frac{(6\ell \chi S_d\kappa )^2}{4\lambda }\Vert \delta \xi _s\Vert _{4}^4. \end{aligned}$$

In a similar way to (3.11), we have

$$\begin{aligned} 12\chi \left\langle |\delta \rho _s|^2 \nabla \delta \rho _s, \nabla \mathcal {G}*\hat{\xi }_s \delta \rho _s \right\rangle \le 2\lambda \Vert \delta \rho _s\nabla \delta \rho _s\Vert _{2}^2 + \frac{(6\ell \chi S_d\kappa )^2}{2\lambda }\Vert \delta \rho _s\Vert _{4}^4. \end{aligned}$$

Thus, combining above estimates gives

3.15 $$\begin{aligned}&12\chi \left\langle |\delta \rho _s|^2 \nabla \delta \rho _s, \nabla \mathcal {G}*\bar{\xi }_s \rho ^{\bar{\xi }}_s- \nabla \mathcal {G}*\hat{\xi }_s \rho ^{\hat{\xi }}_s \right\rangle = 12\chi \left\langle |\delta \rho _s|^2 \nabla \delta \rho _s, \nabla \mathcal {G}*\delta \xi _s \rho ^{\bar{\xi }}_s+ \nabla \mathcal {G}*\hat{\xi }_s \delta \rho _s \right\rangle \nonumber \\&\quad \le 4\lambda \Vert \delta \rho _s\nabla \delta \rho _s\Vert _{2}^2 + \frac{(6\ell \chi S_d\kappa )^2}{\lambda }\Vert \delta \rho _s\Vert _{4}^4+ \frac{(6\ell \chi S_d\kappa )^2}{4\lambda }\Vert \delta \xi _s\Vert _{4}^4, \end{aligned}$$

which together with (3.14) and (3.13) implies

$$\begin{aligned} \Vert \delta \rho _{t}\Vert _{4}^4&\le \left( \frac{(12\varLambda ^2 )^2}{2\lambda }+\frac{(6\ell \chi S_d\kappa )^2}{\lambda }\right) \int _0^t\Vert \delta \rho _s\Vert _{4}^4 \,\mathrm{d}s +\frac{(6\ell \chi S_d\kappa )^2}{4\lambda } \int _0^t \Vert \delta \xi _s\Vert _{4}^4\,\mathrm{d}s\\&\le \frac{4\ln \ell }{T}\int _0^t\Vert \delta \rho _s\Vert _{4}^4 \,\mathrm{d}s +\frac{(6\ell \chi S_d\kappa )^2}{4\lambda } \int _0^t \Vert \delta \xi _s\Vert _{4}^4\,\mathrm{d}s\quad \text {a.s.}. \end{aligned}$$

By Gronwall’s inequality, we get

3.16 $$\begin{aligned} \Vert \delta \rho \Vert _{S_{\mathcal {F}^W}^{\infty }([0,T];L^4(\mathbb {R}^d))}&\le \left[ \frac{(6\ell \chi S_d\kappa )^2T}{4\lambda }\right] ^{\frac{1}{4}}\ell \Vert \delta \xi \Vert _{S_{\mathcal {F}^W}^{\infty }([0,T];L^4(\mathbb {R}^d))}\quad \text {a.s.}. \end{aligned}$$

Hence, whenever $0<\kappa <\kappa _0 \wedge \left[ \frac{4\lambda }{(6\ell ^3\chi S_d)^2T}\right] ^{\frac{1}{2}}$, the solution map $\mathcal T$ is a contraction mapping on the complete metric space $\mathbb B$, and it admits a unique fixed point $\rho =\rho ^{\rho }$ which is the unique solution to SPDE (1.3). $\square$

### Remark 3.1

For the well-posedness of SPDE (1.3), the main difficulty lies in the KS type nonlinear term $-\chi \nabla \cdot ((\nabla \mathcal {G}*\rho _t) \rho _t)$ which prevents us from using the existing methods in the SPDE literature. In view of Eq. (3.8) and the computation that follows, one may see that the stochastic integral there equals zero because of the divergence-free condition (iii) of Assumption 1. This further allows us to obtain $\rho \in S_{\mathcal F^W}^\infty (0,T;L^4(\mathbb {R}^d))$ which finally yields the conclusions in Theorem 3.2 with a *deterministic* $\kappa$. Without (iii) of Assumption 1, one may try to generalize the localization technique with stopping times (see Karatzas and Shreve 1998, Chapter 1, Section 5) for random fields which, however, may incur cumbersome arguments not just for the well-posedness of SPDE (1.3) in this section, but also for the subsequent sections.

In view of the above proof, we can particularly take

$$\begin{aligned} 0< \chi \kappa <\frac{1}{ \ell } \wedge \left[ \frac{4\lambda }{(6\ell ^3 S_d)^2T}\right] ^{\frac{1}{2}},\quad \text {with } \ell = \exp \left\{ \frac{T}{4} \left( \frac{(12\varLambda ^2)^2}{2\lambda }+\frac{(6 S_d)^2}{\lambda }\right) \right\} , \end{aligned}$$

for the well-posedness of SPDE (1.3) in Theorem 3.2. Therefore, whenever $\chi \Vert \rho _0\Vert _{4}<\frac{1}{ \ell } \wedge \left[ \frac{4\lambda }{(6\ell ^3 S_d)^2T}\right] ^{\frac{1}{2}}$, the unique existence of solution in $\mathbb M$ can be asserted as in Theorem 3.2.

Furthermore, suppose that the diffusion coefficients $\nu$ and $\sigma$ are spatial invariant, i.e.,

3.17 $$\begin{aligned} \text {the measurable diffusion coefficients } \sigma ,\,\nu : \left( [0,T],\mathcal B([0,T]\right) \longrightarrow \left( \mathbb {R}^{d\times d'},\mathcal B(\mathbb {R}^{d\times d'})\right) . \end{aligned}$$

Then, the left-hand side of (3.10) and the third term of line (3.14) will vanish. Repeating the proof and combining computations around (3.13) and (3.16), we can obtain the well-posedness of SPDE (1.3) in Theorem 3.2 with a particular selection:

3.18 $$\begin{aligned} 0<\chi \kappa \sqrt{T}<\frac{1}{\ell } \wedge \left[ \frac{4\lambda }{(6\ell ^3 S_d)^2}\right] ^{\frac{1}{2}},\quad \text {with } \ell = \exp \left\{ \frac{(6 S_d)^2}{ 4 \lambda } \right\} , \end{aligned}$$

which indicates that for any given $\rho _0$, the existence and uniqueness of solution may be guaranteed on time interval $[0,T_0]$ if

$$\begin{aligned} T_0< \frac{1}{ \ell ^2 \chi ^2 \Vert \rho _0\Vert _4^2} \wedge \frac{4\lambda }{(6\ell ^3\chi \Vert \rho _0\Vert _4 S_d)^2}. \end{aligned}$$

For this solution on $[0,T_0]$, we may conduct estimates as in the proof of Theorem 3.2. Notice that instead of (3.11) and (3.12), we have

$$\begin{aligned} 12\chi \left\langle \rho _s(\nabla \rho _s),( \nabla \mathcal {G}*\rho _s) \rho _s ^2 \right\rangle&\le 12\chi \Vert \rho _s\nabla \rho _s\Vert _{2} \Vert \rho _s\Vert _{4}^2 \Vert \nabla \mathcal {G}*\rho _s\Vert _{{\infty }} \\&\le 12\chi S_d \Vert \rho _s\nabla \rho _s\Vert _{2} \Vert \rho _s\Vert _{4}^{2}\left\Vert \rho _s \, \right\Vert _4 \\&\le 2\lambda \Vert \rho _s\nabla \rho _s\Vert _{2}^2 + \frac{(6\chi S_d)^2}{2\lambda }\Vert \rho _s\Vert _{4}^{6}, \end{aligned}$$

and

3.19 $$\begin{aligned}&\Vert \rho _{t}\Vert _{4}^4-\Vert \rho _0\Vert _{4}^4\nonumber \\&\quad \le -6\lambda \int _0^{t} \Vert \rho _s\nabla \rho _s\Vert _{2}^2\,\mathrm{d}s +2\lambda \int _0^{t}\Vert \rho _s\nabla \rho _s\Vert _{2}^2\,ds + \frac{(6 \chi S_d)^2}{2\lambda } \int _0^{t} \Vert \rho _s\Vert _{4}^{4} \,\mathrm{d}s \nonumber \\&\quad \le -{\lambda } \int _0^{t} \Vert \nabla (\rho _s^2)\Vert _{2}^2\,\mathrm{d}s + \frac{(6\chi S_d)^2}{2\lambda } \int _0^{t} \Vert \rho _s\Vert _{4}^{6} \,\mathrm{d}s. \end{aligned}$$

Meanwhile, using the Gagliardo–Nirenberg inequality yields that there exists a constant $N_d>0$ depending on *d* such that

$$\begin{aligned} \Vert \rho _s\Vert _4^2=\Vert \rho ^2_s\Vert _2 \le N_d \Vert \nabla (\rho ^2_s)\Vert _2^{\frac{d}{d+2}}\cdot \Vert \rho _s^2\Vert ^{\frac{2}{d+2}}_1&= N_d \Vert \nabla (\rho ^2_s)\Vert _2^{\frac{d}{d+2}}\cdot \Vert \rho _s\Vert ^{\frac{4}{d+2}}_2 \\ \text {(by interpolation inequality) }&\le N_d \Vert \nabla (\rho ^2_s)\Vert _2^{\frac{d}{d+2}}\cdot \Vert \rho _s\Vert ^{\frac{8}{3(d+2)}}_4 \cdot \Vert \rho _s\Vert ^{\frac{4}{3(d+2)}}_1\\&=N_d \Vert \nabla (\rho ^2_s)\Vert _2^{\frac{d}{d+2}}\cdot \Vert \rho _s\Vert ^{\frac{8}{3(d+2)}}_4. \end{aligned}$$

Then, it follows that

$$\begin{aligned} \Vert \nabla (\rho ^2_s)\Vert ^2_2&\ge |N_d|^{-\frac{2(d+2)}{d}} \cdot \Vert \rho _s\Vert _4^{4+\frac{8}{3d}}, \end{aligned}$$

which inserted into (3.19) gives

3.20 $$\begin{aligned} \Vert \rho _{t}\Vert _{4}^4-\Vert \rho _0\Vert _{4}^4 \le&\int _0^{t} \Vert \rho _s\Vert _4^{4+\frac{8}{3d}} \left( \frac{(6\chi S_d)^2}{2\lambda } \cdot \Vert \rho _s\Vert _4^{2-\frac{8}{3d}} -\frac{\lambda }{ |N_d|^{\frac{2(d+2)}{d}}} \right) \,\mathrm{d}s. \end{aligned}$$

Therefore, if

$$\begin{aligned} \frac{(6\chi S_d)^2}{2\lambda } \cdot \Vert \rho _0\Vert _4^{2-\frac{8}{3d}} -\frac{\lambda }{ |N_d|^{\frac{2(d+2)}{d}}} \le 0 \end{aligned}$$,

i.e.,

3.21 $$\begin{aligned} \Vert \rho _0\Vert _4 \le \left( \frac{2\lambda ^2}{ |N_d|^{\frac{2(d+2)}{d}}(6\chi S_d)^2 } \right) ^{\frac{3d}{6d-8}}, \end{aligned}$$

then we conclude from (3.20) that $\Vert \rho _t\Vert _4 \le \Vert \rho _0\Vert _4$ for all $t\in [0,T_0]$ and that the unique solution may actually be extended to any finite time interval [0, *T*].

### Corollary 1

Let Assumption 1 hold with $m=2$ and the diffusion coefficients $\nu$ and $\sigma$ being spatial invariant [see (3.17)]. Assume $0\le \rho _0\in L^1\cap \mathcal {H}^{\frac{1}{2}}_{4}(\mathbb {R}^d)$ with $\left\Vert \rho _0 \, \right\Vert _1=1$. There exists a constant $\kappa >0$ depending only on $\chi ,\lambda ,$ and *d* such that if $\Vert \rho _0\Vert _{4}\le \kappa$, SPDE (1.3) admits a unique nonnegative solution in

$$\begin{aligned} L^2_{\mathcal F^W} (0,T;W^{1,2}(\mathbb {R}^d))\cap L^4_{\mathcal F^W} (0,T;W^{1,4}(\mathbb {R}^d)) \cap S_{\mathcal F^W}^{\infty }([0,T];L^1\cap L^4(\mathbb {R}^d)), \end{aligned}$$

for all $$T>0$$.

In Corollary 1, the constant $\kappa$ may be given as the right-hand side of (3.21) that is independent of $(T,\varLambda )$ and the global solution result with small initial value under $L^4$-norm seems to hold in a similar way as the deterministic counterparts (see Blanchet et al. 2006; Corrias et al. 2004; Biler 2010 for instance). The results in Theorem 3.2, Corollary 1, and subsequent theorems, may be extended to general $L^p$-norms for $p>3$, which would not be discussed in this paper to avoid cumbersome arguments.

To explore the connections between the stochastic Keller–Segel Eq. (1.3) and associated SDEs of McKean–Vlasov type (1.7), we need stronger regularity of the solution.

### Theorem 3.3

Let Assumption 1 hold with $m=3$. Suppose further $\rho _0 \in L^1\cap W^{2,2}(\mathbb {R}^d)$. Then, for any $T>0$, there exists $\kappa >0$ depending only on $T,\varLambda ,\lambda ,\chi$ and *d* such that if $\Vert \rho _0\Vert _{4}\le \kappa$, SPDE (1.3) admits a unique nonnegative solution in

$$\begin{aligned} \mathbb {M}_1:= & {} L^2_{\mathcal F^W} (0,T;W^{3,2}(\mathbb {R}^d)) \cap S_{\mathcal {F}^W}^2 ([0,T];W^{2,2}(\mathbb {R}^d)) \\&\quad \cap ~L^4_{\mathcal {F}^W} (0,T;W^{1,4}(\mathbb {R}^d)) \cap S_{\mathcal {F}^W}^{\infty }([0,T];L^1\cap L^4(\mathbb {R}^d)). \end{aligned}$$

### Proof

Notice that $W^{2,2}(\mathbb {R}^d)\hookrightarrow \mathcal {H}_4^{\frac{1}{2}} (\mathbb {R}^d)\hookrightarrow L^4(\mathbb {R}^d)$ for $d=2$ or 3. Comparing Theorems 3.3 and 3.2, we only need to prove that the obtained unique solution $\rho$ in Theorem 3.2 is also lying in $L^2_{\mathcal F^W} (0,T;W^{3,2}(\mathbb {R}^d)) \cap S_{\mathcal {F}^W}^2 ([0,T];W^{2,2}(\mathbb {R}^d))$. In fact, $\rho \in \mathbb M$ [defined in (3.4)] is the solution of the following linear SPDE:

3.22 $$\begin{aligned} {\left\{ \begin{array}{ll} \,\mathrm {d}\rho _t =\left[ \frac{1}{2}\sum _{i,j=1}^dD_{ij}(\rho _t\sum _{k=1}^{d'}(\nu ^{ik}\nu ^{jk}+ \sigma ^{ik}\sigma ^{jk}))+\chi f_t \right] \, \mathrm{d} t\\ \quad -\sum _{i=1}^dD_i( \rho _t\sum _{k=1}^{d'} \sigma ^{ik})\,\mathrm {d}W_t^k \\ \rho _0\text { is given,} \end{array}\right. } \end{aligned}$$

with

$$\begin{aligned} f_t=-\nabla \cdot (\rho _t\nabla c_t)=-\nabla \rho _t\cdot \nabla c_t+\rho _t^2-\rho _tc_t. \end{aligned}$$

As $\rho \in \mathbb M$, it follows that

$$\begin{aligned} \Vert f_t\Vert _{2}&=\Vert \nabla \cdot (\rho _t\nabla c_t)\Vert _{2} \le \left\Vert \nabla c_t \, \right\Vert _\infty \left\Vert \nabla \rho _t \, \right\Vert _{2}+\Vert \rho _t \Vert _{4}^2+\left\Vert \rho _t \, \right\Vert _2\left\Vert c_t \, \right\Vert _\infty \\&\le S_d \Vert \rho \Vert _{4} \left\Vert \nabla \rho _t \, \right\Vert _{2}+\Vert \rho _t \Vert _{4}^2+S_d\left\Vert \rho _t \, \right\Vert _2\left\Vert \rho _t \, \right\Vert _4\le S_d\Vert \rho \Vert _{4} \left\Vert \rho _t \, \right\Vert _{W^{1,2}}+\Vert \rho _t \Vert _{4}^2 , \end{aligned}$$

which indicates that

3.23 $$\begin{aligned} \Vert f\Vert ^2_{L^2_{\mathcal F^W}(0,T;L^2)} \le 2S_d^2 \Vert \rho \Vert ^2_{S_{\mathcal {F}^W}^{\infty }([0,T];L^4)} \Vert \rho \Vert ^2_{L^2_{\mathcal F^W}(0,T;W^{1,2})}+ 2\Vert \rho \Vert ^4_{L^4_{\mathcal F^W}(0,T;L^4)}<\infty . \end{aligned}$$

The $L^p$-theory of SPDE (see Krylov 1999, Theorem 5.1) and Theorem 3.2 imply that

3.24 $$\begin{aligned} \rho \in L^2_{\mathcal F^W} (0,T;W^{2,2}(\mathbb {R}^d)) \cap S_{\mathcal {F}^W}^2 ([0,T];W^{1,2}(\mathbb {R}^d)) \cap \mathbb M. \end{aligned}$$

Similarly, for $j=1,\dots ,d$, one has

$$\begin{aligned}&\Vert D_jf_t\Vert _{2}\le C\left\Vert \rho _t \, \right\Vert _{W^{2,2}}\left\Vert \rho _t \, \right\Vert _4+C\left\Vert \rho _t \, \right\Vert _{W^{1,4}}\left\Vert \rho _t \, \right\Vert _4, \end{aligned}$$

which together with (3.24) and (3.23) implies that

$$\begin{aligned} \Vert f\Vert _{L^2_{\mathcal F^W}(0,T;W^{1,2})} <\infty . \end{aligned}$$

Hence, applying the $L^p$-theory of SPDE (see Krylov 1999, Theorem 5.1) and Theorem 3.2 again, we conclude

$$\begin{aligned}&\rho \in L^2_{\mathcal F^W} (0,T;W^{3,2}(\mathbb {R}^d)) \cap S_{\mathcal {F}^W}^2 ([0,T];W^{2,2}(\mathbb {R}^d)) \\&\qquad \cap ~L^4_{\mathcal F^W} (0,T;W^{1,4}(\mathbb {R}^d)) \cap S_{\mathcal {F}^W}^{\infty }([0,T];L^1\cap L^4(\mathbb {R}^d)). \end{aligned}$$

The proof is completed. $\square$

## Well-Posedness of the Nonlinear SDE

Let us consider the following SDE:

4.1 $$\begin{aligned} {\left\{ \begin{array}{ll} \,\mathrm {d}Y_t=\chi \nabla \mathcal {G}*\rho _t(Y_t) \, \mathrm{d} t+\nu _t(Y_t) \,\mathrm {d}B_t+\sigma _t(Y_t)\,\mathrm {d}W_t,\quad t>0,\\ \rho _t \text{ is } \text{ the } \text{ conditional } \text{ density } \text{ of } Y_t \text{ given } \mathcal {F}_t^W,\\ Y_0=\zeta ^1, \end{array}\right. } \end{aligned}$$

where we take $B_t=B_t^1$ *in this section* as a $d'$-dimensional Wiener process independent of $W_t$ and $\zeta ^1$. In the following, we prove the well-posedness of the nonlinear SDE (4.1) which actually shares the same solvability as SDE (1.7) for each $i\in \mathbb Z^+$.

### Theorem 4.1

(Well-posedness of the SDE) Under the same assumptions as in Theorem 3.3, let $\rho$ be the regular solution to the SPDE (1.3) obtained in Theorem 3.3. Then, the nonlinear SDE (4.1) has a unique strong solution $(Y_t)_{t\ge 0}$ with $\rho \in S^{2}_{\mathcal {F}^W}([0,T];W^{2,2}(\mathbb {R}^d)) \cap S^{\infty }_{\mathcal {F}^W}([0,T];L^{4}(\mathbb {R}^d))$ being its conditional density under filtration $(\mathcal {F}^W_t)_{t\in [0,T]}$.

### Proof

For the solution $\rho \in S^{2}_{\mathcal {F}^W}(([0,T];W^{2,2}(\mathbb {R}^d)) \cap S^{\infty }_{\mathcal {F}^W}([0,T];L^4(\mathbb {R}^d))$ of the SPDE (1.3) given in Theorem 3.3, by embedding theorems , we have

4.2 $$\begin{aligned}&\nabla \mathcal {G}*\rho \in S_{\mathcal {F}^W}^2([0,T];W^{3,2}(\mathbb {R}^d)) \cap S^{\infty }_{\mathcal {F}^W}([0,T];W^{1,4}(\mathbb {R}^d)) \,\,\nonumber \\&\quad \hookrightarrow \,\,S_{\mathcal {F}^W}^2([0,T];W^{1,\infty }(\mathbb {R}^d)) \cap S^{\infty }_{\mathcal {F}^W}([0,T];L^{\infty }(\mathbb {R}^d)), \end{aligned}$$

which ensures the existence and uniqueness of strong solution $({\overline{Y}}_t)_{t\ge 0}$ to the following linear SDE:

4.3 $$\begin{aligned} {\left\{ \begin{array}{ll} \,\mathrm {d}{\overline{Y}}_t=\chi \nabla \mathcal {G} *\rho _t({\overline{Y}}_t) \, \mathrm{d} t+\nu _t({\overline{Y}}_t) \,\mathrm {d}B_t+\sigma _t({\overline{Y}}_t)\,\mathrm {d}W_t,\quad t>0,\\ {\overline{Y}}_0 =\zeta ^1. \end{array}\right. } \end{aligned}$$

To prove that the conditional density given $\mathcal {F}_t^W$ of $({\overline{Y}}_t)_{t\ge 0}$ exists and is the solution to SPDE (1.3), we need the following result on backward SPDE and associated probabilistic representation.

### Lemma 4.1

Let Assumption 1 hold with $m=3$, $\rho \in S^{2}_{\mathcal {F}^W}([0,T];W^{2,2}(\mathbb {R}^d)) \cap S^{\infty }_{\mathcal {F}^W}([0,T];L^{4}(\mathbb {R}^d))$ and $T_1\in (0,T]$. Then, for each $G\in L^2(\Omega , \mathcal {F}_{T_1}; W^{2,2}(\mathbb {R}^d))$, the following backward SPDE:

4.4 $$\begin{aligned} \left\{ \begin{aligned}&-\,\mathrm {d}u_t =\left[ \frac{1}{2}\sum _{i,j=1}^d\sum _{k=1}^{d'}(\nu ^{ik}_t\nu ^{j,k}_t+ \sigma ^{ik}_t\sigma ^{jk}_t)D_{ij}u_t +\chi \sum _{i=1}^d D_i\mathcal {G} *\rho _t D_iu_t + \sum _{i=1}^d\sum _{k=1}^{d'} \sigma _t^{ik}D_i \psi _t^k \right] \,\mathrm{d}t \\&-\sum _{k=1}^{d'} \psi _t^k \,\mathrm {d}W_t^k, \\&u_{T_1}=G , \end{aligned} \right. \end{aligned}$$

admits a unique solution

$$\begin{aligned} (u,\psi ) \in \left( L^2_{\mathcal F^W} (0,T;W^{3,2}(\mathbb {R}^d)) \cap S_{\mathcal {F}^W}^2 ([0,T];W^{2,2}(\mathbb {R}^d)) \right) \times L^2_{\mathcal F^W} (0,T;W^{2,2}(\mathbb {R}^d)) , \end{aligned}$$

i.e., for any $\varphi \in C_c^2(\mathbb {R}^d)$, there holds for each $t\in [0,T_1]$,

$$\begin{aligned} \langle u_t, \,\varphi \rangle&= \langle \varphi ,\,G\rangle \\&\qquad +\int _t^{T_1} \bigg \langle \varphi ,\, \frac{1}{2}\sum _{i,j=1}^d\sum _{k=1}^{d'}(\nu _s^{ik}\nu _s^{jk}+ \sigma _s^{ik}\sigma _s^{jk})D_{ij}u_s +\chi \sum _{i=1}^d D_i\mathcal {G} *\rho _s D_iu_s + \sum _{i=1}^d\sum _{k=1}^{d'} \sigma _s^{ik}D_i \psi _s^k \bigg \rangle \,\mathrm{d}s \\&\qquad -\int _t^{T_1} \sum _{k=1}^{d'} \left\langle \varphi ,\, \psi ^k_s\right\rangle \,\mathrm{d}W_s^k ,\quad \text {a.s.} \end{aligned}$$

Moreover, for this solution, we have

4.5 $$\begin{aligned} u_t(y)=\mathbb {E}\left[ G({\overline{Y}}_{T_1}) \big |{\overline{Y}}_t=y,\, \mathcal {F}_t^W\right] ,\quad \mathrm {a.s. for any }t\in [0,T_1]. \end{aligned}$$

For each $T_1\in (0,T]$, take an arbitrary $\xi \in L^{\infty }(\Omega ,\mathcal {F}_{T_1})$ and $\phi \in C_c^{\infty }(\mathbb {R}^d)$. In view of the SPDE (1.3), applying the Itô formula to $\langle u_t,\,\rho _t\rangle$ [the duality analysis on the (1.3) and (4.4) as in Du et al. (2011), Zhou (1992)] gives

$$\begin{aligned} \langle u_0, \rho _0\rangle = \langle \xi \phi , \rho _{T_1} \rangle -\int _0^{T_1} \sum _{i=1}^d\sum _{k=1}^{d'} \langle u_t,\,D_i(\sigma ^{ik}_t\rho _t)\rangle \, \mathrm{d}W^k_t -\int _0^{T_1} \sum _{k=1}^{d'} \langle \rho _t,\, \psi ^k_t\rangle \, \mathrm{d}W^k_t, \quad \text {a.s.,} \end{aligned}$$

where $(u,\psi )$ is the solution in Lemma 4.1 with $G=\xi \phi$. Then, we have by taking expectations on both sides,

$$\begin{aligned} \langle u_0, \rho _0\rangle = \mathbb {E}[\langle \xi \phi , \rho _{T_1} \rangle ] = \mathbb {E}[\xi \langle \phi , \rho _{T_1} \rangle ] . \end{aligned}$$

On the other hand, in view of the probabilistic representation (4.5), we have

$$\begin{aligned} \langle u_0, \rho _0\rangle =\int _{\mathbb {R}^d}\mathbb {E}\left[ G(\overline{Y}_{T_1}) \big |{\overline{Y}}_0=y,\, \mathcal {F}_0^W\right] \rho _0(y) \, \mathrm{d} y=\mathbb {E}[\xi \phi ({\overline{Y}}_{T_1}) ]= \mathbb {E}\left[ \xi \mathbb {E}[\phi (\overline{Y}_{T_1}) \big | \mathcal {F}^W_{T_1}] \right] . \end{aligned}$$

Therefore,

$$\begin{aligned} \mathbb {E}[\xi \langle \phi , \rho _{T_1} \rangle ] = \mathbb {E}\left[ \xi \mathbb {E}[\phi ({\overline{Y}}_{T_1}) \big | \mathcal {F}^W_{T_1}] \right] , \end{aligned}$$

which by the arbitrariness of $(T_1,\xi ,\phi )$ implies that $\rho _t$ is the conditional density of ${\overline{Y}}_t$ given $\mathcal {F}_t^W$ for each $t\in [0,T]$ and shows the existence of strong solution to SDE (4.1). In fact, this also means that each strong solution of SDE (4.1) with $\rho \in S^{2}_{\mathcal {F}^W}([0,T];W^{2,2}(\mathbb {R}^d)) \cap S^{\infty }_{\mathcal {F}^W}([0,T];L^{4}(\mathbb {R}^d))$ must have the conditional density $\rho$ being the solution to SPDE (1.3), and thus, the strong solution is unique. We complete the proof. $\square$

### Proof of Lemma 4.1

Embedding theorem gives (4.2) which by the $L^2$-theory of backward SPDE (see Du et al. 2011; Zhou 1992) implies that backward SPDE (4.4) has a unique solution $(u,\psi )\in \left( L^2_{\mathcal F^W} (0,T;W^{1,2}(\mathbb {R}^d)) \cap S_{\mathcal {F}^W}^2 ([0,T];L^2(\mathbb {R}^d)) \right) \times L^2_{\mathcal F^W} (0,T;L^2(\mathbb {R}^d))$.^4^ Then, we need to show that the solution $(u,\psi )$ has higher regularity as it is done in the proof of Theorem 3.3. In fact, we have for each $i=1,\dots ,d$,

$$\begin{aligned} \left\| D_i\mathcal {G} *\rho _s D_iu_s \right\| _2 \le \left\| D_i\mathcal {G} *\rho _s \right\| _{\infty } \left\| D_iu_s \right\| _2 \le S_d\Vert \rho _s\Vert _4 \left\| D_iu_s \right\| _2, \end{aligned}$$

and thus, $D_i\mathcal {G} *\rho D_iu \in L^2_{\mathcal {F}^W} (0,T_1;L^2)$, which by $L^2$-theory of backward SPDE indicated further

4.6 $$\begin{aligned} (u,\psi )\in \left( L^2_{\mathcal F^W} (0,T;W^{2,2}(\mathbb {R}^d)) \cap S_{\mathcal {F}^W}^2 ([0,T];W^{1,2}(\mathbb {R}^d)) \right) \times L^2_{\mathcal F^W} (0,T;W^{1,2}(\mathbb {R}^d)). \end{aligned}$$

Taking derivatives gives further

$$\begin{aligned} \left\| D_j(D_i\mathcal {G} *\rho _s D_iu_s) \right\| _2&\le \left\| D_{ij}\mathcal {G} *\rho _s D_iu_s \right\| _2 + \left\| D_{i}\mathcal {G} *\rho _s D_{ij}u_s \right\| _2 \\&\le \left\| D_{ij}\mathcal {G} *\rho _s\right\| _4\left\| D_iu_s \right\| _4 + \left\| D_{i}\mathcal {G} *\rho _s\right\| _{\infty } \left\| D_{ij}u_s \right\| _2 \\&\le S_d\left\| \rho _s\right\| _4 \left\| D_iu_s \right\| _2^{1/4} \left\| D_iu_s \right\| _6^{3/4} + S_d\left\| \rho _s\right\| _{4} \left\| D_{ij}u_s \right\| _2 \\&\le \left\| \rho _s\right\| _4 \left\| u_s \right\| _{W^{2,2}} + S_d\left\| \rho _s\right\| _{4} \left\| u_s \right\| _{W^{2,2}}, \end{aligned}$$

and thus, $D_i\mathcal {G} *\rho _s D_iu_s \in L^2_{\mathcal {F}^W} (0,T_1;W^{1,2}(\mathbb {R}^d))$, $i=1,\dots ,d$. Applying the $L^2$-theory again, we arrive at

$$\begin{aligned} (u,\psi ) \in \left( L^2_{\mathcal F^W} (0,T;W^{3,2}(\mathbb {R}^d)) \cap S_{\mathcal {F}^W}^2 ([0,T];W^{2,2}(\mathbb {R}^d)) \right) \times L^2_{\mathcal F^W} (0,T;W^{2,2}(\mathbb {R}^d)) . \end{aligned}$$

W.l.o.g., we prove the probabilistic representation (4.5) for the case when $t=0$. In fact, a straightforward application of Yang and Tang (2013, Theorem 3.1) yields that

$$\begin{aligned} u_0(y)=G({\overline{Y}}_{T_1}) -\int _0^{T_1} \left( \sum _k^{d'} \psi ^k_s({\overline{Y}}_s) + \sum _{i=1}^d\sigma ^{ik}_s(\overline{Y}_s) D_iu_s({\overline{Y}}_s)\right) \,\mathrm{d}W^k_s, \quad \text {a.s.} \end{aligned}$$

Noticing that by embedding theorem it holds that $L^2_{\mathcal F^W} (0,T;W^{2,2}(\mathbb {R}^d)) \,\, \hookrightarrow \,\, L^2_{\mathcal F^W} (0,T;C^{1/4}(\mathbb {R}^d))$, we may easily check that the stochastic integral in the above equality is mean-zero. Therefore, we have $u_0(y)=\mathbb {E}\left[ G({\overline{Y}}_{T_1}) \big |{\overline{Y}}_0=y,\, \mathcal {F}_0^W\right]$ by taking conditional expectation on both sides. For general $t\in (0,T_1]$, the proof of (4.5) follows similarly. $\square$

##  Mean-Field Limit of the Particle System (1.5) Toward the Stochastic KS Equation (1.3)

To prove the mean-field limit, we recall the following auxiliary stochastic dynamics $\{(Y_t^i)_{t\ge 0}\}_{i=1}^N$ as defined in (1.7)

5.1 $$\begin{aligned} {\left\{ \begin{array}{ll} \,\mathrm {d}Y_t^i= \chi \nabla \mathcal {G}*\rho _t(Y_t^i) \, \mathrm{d} t+\nu _t(Y_t^i) \,\mathrm {d}B_t^i+\sigma _t(Y_t^i)\,\mathrm {d}W_t,\quad t>0,\quad i=1,\ldots ,N,\\ \rho _t \text{ is } \text{ the } \text{ conditional } \text{ density } \text{ of } Y_t^i \text{ given } \mathcal {F}_t^W \text{ for } \text{ all } i=1,\ldots ,N. \\ Y_0^i=\zeta ^i. \end{array}\right. } \end{aligned}$$

This means that $\{(Y_t^i)_{t\ge 0}\}_{i=1}^N$ are *N* copies of solutions to the nonlinear SDE (4.1), and they are conditional i.i.d. given $W_t$. We will also use the regularized version

5.2 $$\begin{aligned} {\left\{ \begin{array}{ll} \,\mathrm {d}Y_t^{i,\varepsilon }= \chi \nabla \mathcal {G}_\varepsilon *\rho _t^\varepsilon (Y_t^{i,\varepsilon }) \, \mathrm{d} t+\nu _t(Y_t^{i,\varepsilon }) \,\mathrm {d}B_t^i+\sigma _t(Y_t^{i,\varepsilon })\,\mathrm {d}W_t,\quad t>0,\quad i=1,\ldots ,N,\\ \rho _t^\varepsilon \text{ is } \text{ the } \text{ conditional } \text{ density } \text{ of } Y_t^{i,\varepsilon } \text{ given } \mathcal {F}_t^W \text{ for } \text{ all } i=1,\ldots ,N. \\ Y_0^{i,\varepsilon }=Y_0^i=\zeta ^i, \end{array}\right. } \end{aligned}$$

with $\rho _t^\varepsilon$ satisfying the following regularized stochastic KS equation

5.3 $$\begin{aligned} {\left\{ \begin{array}{ll} \,\mathrm {d}\rho _t^\varepsilon =&{}\frac{1}{2}\sum _{i,j=1}^dD_{ij}\left( \rho _t^\varepsilon \sum _{k=1}^{d'}(\nu ^{ik}_t\nu ^{jk}_t+ \sigma ^{ik}_t\sigma ^{jk}_t)\right) \, \mathrm{d} t-\chi \nabla \cdot (\nabla (\mathcal {G}_\varepsilon *\rho _t^\varepsilon )\rho _t^\varepsilon ) \, \mathrm{d} t\\ &{}-\sum _{i=1}^{d}D_i\left( \rho _t^\varepsilon \sum _{k=1}^{d'}\sigma ^{ik}_t\,\mathrm {d}W_t^k\right) , \\ \rho _0^\varepsilon =\rho _0, \end{array}\right. } \end{aligned}$$

Indeed, following the same arguments as in Sects. 3–4, we obtain the well-posedness of the regularized system (5.2) and Eq. (5.3). Next, we estimate the difference of the solutions. Set $e_t^\varepsilon =\rho _t^\varepsilon -\rho _t$ for $t\in [0,T]$ with $e_0^\varepsilon =0$. Following the same computation as in (3.14), one has

5.4 $$\begin{aligned} \Vert e_t^\varepsilon \Vert _{4}^4&\le -6\lambda \int _0^{t} \Vert e_s^\varepsilon \nabla e_s^\varepsilon \Vert _2^2\,\mathrm{d}s +2 \lambda \int _0^t\Vert e_s^\varepsilon \nabla e_s^\varepsilon \Vert _{2}^2\,\mathrm{d}s+\frac{(12\varLambda ^2 )^2}{2\lambda }\int _0^t\Vert e_s^\varepsilon \Vert _{4}^4\,\mathrm{d}s \nonumber \\&\qquad +\int _0^t12\chi \left\langle |e_s^\varepsilon |^2 \nabla e_s^\varepsilon , (\nabla \mathcal {G}_\varepsilon *\rho _s^\varepsilon )\rho _s^\varepsilon - (\nabla \mathcal {G}*\rho _s) \rho _s \right\rangle \,\mathrm{d}s,\quad \text {a.s.}. \end{aligned}$$

Notice that

$$\begin{aligned}&12\chi \left\langle |e_s^\varepsilon |^2 \nabla e_s^\varepsilon , (\nabla \mathcal {G}_\varepsilon *\rho _s^\varepsilon )\rho _s^\varepsilon - (\nabla \mathcal {G}*\rho _s) \rho _s \right\rangle \\&\quad = 12\chi \left\langle |e_s^\varepsilon |^2 \nabla e_s^\varepsilon , \nabla \mathcal {G}*e_s^\varepsilon \rho ^{\varepsilon }_s+ \nabla \mathcal {G}*\rho _s e_s^\varepsilon \right\rangle +12\chi \left\langle |e_s^\varepsilon |^2 \nabla e_s^\varepsilon , (\nabla \mathcal {G}_\varepsilon -\nabla \mathcal {G})*\rho _s^\varepsilon \rho _s^\varepsilon \right\rangle . \end{aligned}$$

Similar to the computation in (3.15), one obtains

$$\begin{aligned} 12\chi \left\langle |e_s^\varepsilon |^2 \nabla e_s^\varepsilon , \nabla \mathcal {G}*e_s^\varepsilon \rho ^{\varepsilon }_s+ \nabla \mathcal {G}*\rho _s e_s^\varepsilon \right\rangle \le 2\lambda \Vert e_s^\varepsilon \nabla e_s^\varepsilon \Vert _{2}^2 +C(T,\chi ,\lambda ,\varLambda ,d)\Vert e_s^\varepsilon \Vert _{4}^4. \end{aligned}$$

On the other hand, we compute

$$\begin{aligned} 12\chi \left\langle |e_s^\varepsilon |^2 \nabla e_s^\varepsilon , (\nabla \mathcal {G}_\varepsilon -\nabla \mathcal {G})*\rho _s^\varepsilon \rho _s^\varepsilon \right\rangle \le 12\chi \Vert e_s^\varepsilon \nabla e_s^\varepsilon \Vert _{2}\left\Vert (\nabla \mathcal {G}_\varepsilon -\nabla \mathcal {G})*\rho _s^\varepsilon \, \right\Vert _\infty \Vert e_s^\varepsilon \Vert _4\Vert \rho _s^\varepsilon \Vert _4. \end{aligned}$$

Notice that

5.5 $$\begin{aligned}&|(\nabla \mathcal {G}_\varepsilon -\nabla \mathcal {G})*\rho _s^\varepsilon |(x) \nonumber \\&\quad = |\psi _\varepsilon *(\nabla \mathcal {G}*\rho _s^\varepsilon )-\nabla \mathcal {G}*\rho _s^\varepsilon |(x)=\left| \int _{{\mathbb R}^d}\psi _\varepsilon (y)[\nabla \mathcal {G}*\rho _s^\varepsilon (x-y)-\nabla \mathcal {G}*\rho _s^\varepsilon (x)] \, \mathrm{d} y\right| \nonumber \\&\quad \le \left\Vert \nabla \mathcal {G}*\rho _s^\varepsilon \, \right\Vert _{W^{1,\infty }}\int _{\mathbb {R}^d}|y|\psi _\varepsilon (y) \, \mathrm{d} y\le C\varepsilon \left\Vert \nabla \mathcal {G}*\rho _s^\varepsilon \, \right\Vert _{W^{1,\infty }}\le C\varepsilon \left\Vert \rho _s^\varepsilon \, \right\Vert _{W^{1,4}}, \end{aligned}$$

where *C* depends only on $T,\chi ,\lambda ,\varLambda ,$ and *d*. Then, one has

5.6 $$\begin{aligned} 12\chi \left\langle |e_s^\varepsilon |^2 \nabla e_s^\varepsilon , (\nabla \mathcal {G}_\varepsilon -\nabla \mathcal {G})*\rho _s^\varepsilon \rho _s^\varepsilon \right\rangle \le 2 \lambda \Vert e_s^\varepsilon \nabla e_s^\varepsilon \Vert _{2}^2+C\varepsilon ^2\left\Vert \rho _s^\varepsilon \, \right\Vert _{W^{1,4}}^2\Vert e_s^\varepsilon \Vert _4^2\Vert \rho _s^\varepsilon \Vert _4^2,\nonumber \\ \end{aligned}$$

and thus

5.7 $$\begin{aligned}&12\chi \left\langle |e_s^\varepsilon |^2 \nabla e_s^\varepsilon , (\nabla \mathcal {G}_\varepsilon *\rho _s^\varepsilon )\rho _s^\varepsilon - (\nabla \mathcal {G}*\rho _s) \rho _s \right\rangle \nonumber \\&\quad \le 4\lambda \Vert e_s^\varepsilon \nabla e_s^\varepsilon \Vert _{2}^2+C\varepsilon ^2\left\Vert \rho _s^\varepsilon \, \right\Vert _{W^{1,4}}^2\Vert e_s^\varepsilon \Vert _4^2\Vert \rho _s^\varepsilon \Vert _4^2+C\left\Vert e_s^\varepsilon \, \right\Vert _4^4. \end{aligned}$$

It follows from (5.4) that

$$\begin{aligned} \Vert e_t^\varepsilon \Vert _{4}^4 \le C_1\int _0^t\Vert e_s^\varepsilon \Vert _4^4\, \mathrm{d} s+C_2\varepsilon ^2\int _0^t\left\Vert \rho _s^\varepsilon \, \right\Vert _{W^{1,4}}^2\Vert e_s^\varepsilon \Vert _4^2\Vert \rho _s^\varepsilon \Vert _4^2\, \mathrm{d} s, \end{aligned}$$

where $C_1,C_2$ depend only on $T,\chi ,\lambda ,\varLambda$, and *d*. By Gronwall’s inequality, we have

5.8 $$\begin{aligned} \sup _{t\in [0,T]}\Vert e_t^\varepsilon \Vert _{4}^4\le \left[ C_2\varepsilon ^2 \sup _{t\in [0,T]}\{\Vert e_t^\varepsilon \Vert _4^2\Vert \rho _t^\varepsilon \Vert _4^2\}\int _0^T\left\Vert \rho _s^\varepsilon \, \right\Vert _{W^{1,4}}^2\, \mathrm{d} s\right] \exp (C_1T)\quad \text {a.s.}.\nonumber \\ \end{aligned}$$

This leads to

5.9 $$\begin{aligned}&\Vert \rho ^\varepsilon -\rho \Vert _{S_{\mathcal {F}^W}^{2}([0,T];L^4(\mathbb {R}^d))} \nonumber \\&\quad \le C\left( C_1,C_2,T,\Vert \rho \Vert _{S_{\mathcal {F}^W}^{\infty }([0,T];L^4(\mathbb {R}^d))},\Vert \rho ^\varepsilon \Vert _{S_{\mathcal {F}^W}^{\infty }([0,T];L^4(\mathbb {R}^d))},\Vert \rho ^\varepsilon \Vert _{L_{\mathcal {F}^W}^{2}([0,T];W^{1,4}(\mathbb {R}^d))}\right) \varepsilon \nonumber \\&\quad \le C\left( T, \chi ,\lambda ,\varLambda ,d\right) \cdot \varepsilon \end{aligned}$$

where we have used the fact that the quantities $\Vert \rho \Vert _{S_{\mathcal {F}^W}^{\infty }([0,T];L^4(\mathbb {R}^d))},\Vert \rho ^\varepsilon \Vert _{S_{\mathcal {F}^W}^{\infty }([0,T];L^4(\mathbb {R}^d))}$, and $\Vert \rho ^\varepsilon \Vert _{L_{\mathcal {F}^W}^{2}([0,T];W^{1,4}(\mathbb {R}^d))}$ depend only on $T,\chi ,\lambda ,\varLambda$, and *d*, independent of $\varepsilon$.

Our main theorem of mean-field limit states that the mean-field dynamics $\{(Y_t^{i,\varepsilon })_{t\ge 0}\}_{i=1}^N$ well approximate the regularized interacting particle system $\{(X_t^{i,\varepsilon })_{t\ge 0}\}_{i=1}^N$ in (1.5).

### Theorem 5.1

Under the same assumptions as in Theorem 3.3, let $\{(X_t^{i,\varepsilon })_{t\ge 0}\}_{i=1}^N$ and $\{(Y_t^{i,\varepsilon })_{t\ge 0}\}_{i=1}^N$ satisfy the interacting particle system (1.5) and the mean-field dynamics (5.2), respectively. Then, for any fixed $0<\delta \ll 1$, such that $\varepsilon ^{-d}\le \delta \ln (N)$ and $C\delta <1$ it holds that

5.10 $$\begin{aligned} \sup \limits _{t\in [0,T]}\sup \limits _{i=1,\ldots ,N}\mathbb {E}\left[ \left| X_t^{i,\varepsilon }-Y_t^{i,\varepsilon }\right| ^2\right] \le C\frac{(\delta \ln (N))^{\frac{2d-2}{d}} }{N^{1-C\delta }}, \end{aligned}$$

where *C* is a constant depending only on $\chi ,T,d,d'$ and $\varLambda$.

### Proof

Applying Itô’s formula yields that

$$\begin{aligned}&|X_t^{i,\varepsilon }-Y_t^{i,\varepsilon }|^2\\&\quad = \int _0^t 2\chi (X_s^{i,\varepsilon }-Y_s^{i,\varepsilon })\cdot \left( \frac{1}{N-1}\sum \limits _{j\ne i}^N \nabla \mathcal {G}_\varepsilon (X_s^{i,\varepsilon }-X_s^{j,\varepsilon })- \nabla \mathcal {G}_\varepsilon *\rho _s^{\varepsilon }(Y_s^{i,\varepsilon })\right) \, \mathrm{d} s\\&\qquad +\int _0^t2(X_s^{i,\varepsilon }-Y_s^{i,\varepsilon })\cdot (\nu _s(X_s^{i,\varepsilon })-\nu _s(Y_s^{i,\varepsilon })) \,\mathrm {d}B_s^i\\&\qquad +\int _0^t2(X_s^{i,\varepsilon }-Y_s^{i,\varepsilon })\cdot (\sigma _s(X_s^{i,\varepsilon })-\sigma _s(Y_s^{i,\varepsilon }))\,\mathrm {d}W_s \\&\qquad +\int _0^t\sum _j^d\sum _{k=1}^{d'}\left( \nu ^{jk}_s(X_s^{i,\varepsilon })-\nu ^{jk}_s(Y_s^{i,\varepsilon })\right) ^2\, \mathrm{d} s\\&\qquad +\int _0^t\sum _j^d\sum _{k=1}^{d'}\left( \sigma ^{jk}_s(X_s^{i,\varepsilon })-\sigma ^{jk}_s(Y_s^{i,\varepsilon })\right) ^2\, \mathrm{d} s. \end{aligned}$$

Taking expectations on both sides, one has

5.11 $$\begin{aligned}&\mathbb {E}\left[ |X_t^{i,\varepsilon }-Y_t^{i,\varepsilon }|^2\right] \nonumber \\&\quad \le \mathbb {E}\left[ \int _0^t 2\chi (X_s^{i,\varepsilon }-Y_s^{i,\varepsilon })\cdot \left( \frac{1}{N-1}\sum \limits _{j\ne i}^N \nabla \mathcal {G}_\varepsilon (X_s^{i,\varepsilon }-X_s^{j,\varepsilon })- \nabla \mathcal {G}_\varepsilon *\rho _s^{\varepsilon }(Y_s^{i,\varepsilon })\right) \, \mathrm{d} s\right] \nonumber \\&\qquad +C(d,d',\varLambda )\int _0^t\mathbb {E}\left[ |X_s^{i,\varepsilon }-Y_s^{i,\varepsilon }|^2\right] \, \mathrm{d} s, \end{aligned}$$

where we have used the fact that

$$\begin{aligned}&\mathbb {E}\left[ \int _0^t2(X_s^{i,\varepsilon }-Y_s^{i,\varepsilon })\cdot (\nu _s(X_s^{i,\varepsilon })-\nu _s(Y_s^{i,\varepsilon })) \,\mathrm {d}B_s^i\right] \\&\quad =\mathbb {E}\left[ \int _0^t2(X_s^{i,\varepsilon }-Y_s^{i,\varepsilon })\cdot (\sigma _s(X_s^{i,\varepsilon })-\sigma _s(Y_s^{i,\varepsilon }))\,\mathrm {d}W_s\right] =0, \end{aligned}$$

and (ii) in Assumption 1.

To continue, we split the error

$$\begin{aligned} \mathbb {E}\left[ \int _0^t 2\chi (X_s^{i,\varepsilon }-Y_s^{i,\varepsilon })\cdot \left( \frac{1}{N-1}\sum \limits _{j\ne i}^N \nabla \mathcal {G}_\varepsilon (X_s^{i,\varepsilon }-X_s^{j,\varepsilon })- \nabla \mathcal {G}_\varepsilon *\rho _s^{\varepsilon }(Y_s^{i,\varepsilon })\right) \, \mathrm{d} s\right] \end{aligned}$$

into three parts. Notice that

$$\begin{aligned}&\frac{1}{N-1}\sum \limits _{j\ne i}^N \nabla \mathcal {G}_\varepsilon (X_s^{i,\varepsilon }-X_s^{j,\varepsilon })- \nabla \mathcal {G}_\varepsilon *\rho _s^{\varepsilon }(Y_s^{i,\varepsilon })\\&\quad = \frac{1}{N-1} \left( \sum \limits _{j\ne i}^N \nabla \mathcal {G}_\varepsilon (X_s^{i,\varepsilon }-X_s^{j,\varepsilon })- \sum \limits _{j\ne i}^N \nabla \mathcal {G}_\varepsilon (Y_s^{i,\varepsilon }-Y_s^{j,\varepsilon })\right) \\&\qquad +\frac{1}{N-1} \sum \limits _{j\ne i}^N \nabla \mathcal {G}_\varepsilon (Y_s^{i,\varepsilon }-Y_s^{j,\varepsilon })- \nabla \mathcal {G}_\varepsilon *\rho _s^{\varepsilon }(Y_s^{i,\varepsilon })\\&\quad =:I_{11}^s+I_{12}^s. \end{aligned}$$

First, we compute

$$\begin{aligned} \int _0^t 2\chi (X_s^{i,\varepsilon }-Y_s^{i,\varepsilon })\cdot I_{11}^s \, \mathrm{d} s&\le 2\chi \int _0^t |X_s^{i,\varepsilon }-Y_s^{i,\varepsilon }| \frac{1}{N-1}\left\Vert \nabla \mathcal {G}_\varepsilon \, \right\Vert _{W^{1,\infty }}\sum \limits _{j=1}^N\left| X_s^{j,\varepsilon }-Y_s^{j,\varepsilon } \right| \, \mathrm{d} s\\&\le \frac{C\varepsilon ^{-d}}{N-1}\int _0^t\sum \limits _{j=1}^N\left| X_s^{j,\varepsilon }-Y_s^{j,\varepsilon } \right| ^2\, \mathrm{d} s, \end{aligned}$$

which leads to

5.12 $$\begin{aligned} \mathbb {E}\left[ \int _0^t 2\chi (X_s^{i,\varepsilon }-Y_s^{i,\varepsilon })\cdot I_{11}^s \, \mathrm{d} s\right]&\le \frac{C\varepsilon ^{-d}}{N-1}\int _0^t\sum \limits _{j=1}^N\mathbb {E}\left[ \left| X_s^{j,\varepsilon }-Y_s^{j,\varepsilon } \right| ^2\right] \, \mathrm{d} s\nonumber \\&\le C\varepsilon ^{-d}\int _0^t\sup \limits _{i=1,\ldots ,N}\mathbb {E}\left[ \left| X_s^{i,\varepsilon }-Y_s^{i,\varepsilon }\right| ^2\right] \, \mathrm{d} s, \end{aligned}$$

where *C* depends only on $\chi$ and *d*.

To estimate the second term, we rewrite

$$\begin{aligned} I_{12}^s= \frac{1}{N-1}\sum \limits _{j\ne i}^N\left( \nabla \mathcal {G}_\varepsilon (Y_s^{i,\varepsilon }-Y_s^{j,\varepsilon })- \nabla \mathcal {G}_\varepsilon *\rho _s^{\varepsilon }(Y_s^{i,\varepsilon })\right) =:\frac{1}{N-1}\sum \limits _{j\ne i}^NZ_j^i, \end{aligned}$$

where

$$\begin{aligned} Z_j^i=\nabla \mathcal {G}_\varepsilon (Y_s^{i,\varepsilon }-Y_s^{j,\varepsilon })- \nabla \mathcal {G}_\varepsilon *\rho _s^{\varepsilon }(Y_s^{i,\varepsilon }),\quad j\ne i. \end{aligned}$$

It is easy to check that

$$\begin{aligned} \mathbb {E}[Z_j^i|\mathcal {F}_t^W,Y_s^{i,\varepsilon }]=\nabla \mathcal {G}_\varepsilon *\rho _s^{\varepsilon }(Y_s^{i,\varepsilon })- \nabla \mathcal {G}_\varepsilon *\rho _s^{\varepsilon }(Y_s^{i,\varepsilon })=0, \end{aligned}$$

since $\{Y_s^{j,\varepsilon }\}_{j=1}^N$ are conditional i.i.d. with common conditional density $\rho _s^{\varepsilon }$ given $\mathcal {F}_t^W$. Thus, one concludes that

$$\begin{aligned} \mathbb {E}[|I_{12}^s|^2]&=\frac{1}{(N-1)^2}\mathbb {E}\left[ \left( \sum \limits _{j\ne i}^NZ_j^i\right) \left( \sum \limits _{k\ne i}^NZ_k^i\right) \right] \\&=\frac{1}{(N-1)^2}\mathbb {E}\left[ \mathbb {E}\left[ \left( \sum \limits _{j\ne i}^NZ_j^i\right) \left( \sum \limits _{k\ne i}^NZ_k^i\right) |\mathcal {F}_t^W,Y_s^{i,\varepsilon }\right] \right] \\&=\frac{1}{(N-1)^2}\mathbb {E}\left[ \mathbb {E}\left[ \sum \limits _{j\ne i}^N|Z_j^i|^2|\mathcal {F}_t^W,Y_s^{i,\varepsilon }\right] \right] =\frac{1}{N-1}\mathbb {E}[|Z_{1}^2|^2]. \end{aligned}$$

Due to the fact that, using (2.3),

$$\begin{aligned} \mathbb {E}[|Z_{2}^1|^2]=\mathbb {E}\left[ (\nabla \mathcal {G}_\varepsilon (Y_s^{1,\varepsilon }-Y_s^{2,\varepsilon })- \nabla \mathcal {G}_\varepsilon *\rho _s^{\varepsilon }(Y_s^{1,\varepsilon }))^2\right] \le 4\Vert \nabla \mathcal {G}_\varepsilon \Vert _\infty ^2\le C\varepsilon ^{-2(d-1)}, \end{aligned}$$

one has

$$\begin{aligned} \mathbb {E}[|I_{12}^s|^2]\le \frac{C\varepsilon ^{-2(d-1)}}{N-1}. \end{aligned}$$

Thus, we concludes

$$\begin{aligned}&\mathbb {E}\left[ \int _0^t 2\chi (X_s^{i,\varepsilon }-Y_s^{i,\varepsilon })\cdot I_{12}^s \, \mathrm{d} s\right] \\&\quad \le \int _0^t \mathbb {E}\left[ |X_s^{i,\varepsilon }-Y_s^{i,\varepsilon }|^2\right] \, \mathrm{d} s+\int _0^t\chi ^2 \mathbb {E}\left[ |I_{12}^s|^2\right] \, \mathrm{d} s\le \int _0^t\mathbb {E}\left[ |X_s^{i,\varepsilon }-Y_s^{i,\varepsilon }|^2\right] \, \mathrm{d} s\\&\qquad +\frac{C\varepsilon ^{-2(d-1)}}{N-1}, \end{aligned}$$

where *C* depends only on $\chi$ and *d*.

Now, collecting estimates (5.12) and (5.13) implies

5.13 $$\begin{aligned}&\mathbb {E}\left[ \int _0^t 2\chi (X_s^{i,\varepsilon }-Y_s^{i,\varepsilon })\cdot \left( \frac{1}{N-1}\sum \limits _{j\ne i}^N \nabla \mathcal {G}_\varepsilon (X_s^{i,\varepsilon }-X_s^{j,\varepsilon })- \nabla \mathcal {G}_\varepsilon *\rho _s^{\varepsilon }(Y_s^{i,\varepsilon })\right) \, \mathrm{d} s\right] \nonumber \\&\quad \le C\varepsilon ^{-d}\int _0^t\sup \limits _{i=1,\ldots ,N}\mathbb {E}\left[ \left| X_s^{i,\varepsilon }-Y_s^{i,\varepsilon }\right| ^2\right] \, \mathrm{d} s+\frac{C\varepsilon ^{-2(d-1)}}{N-1} \end{aligned}$$

which together with (5.11) lead to

$$\begin{aligned} \sup \limits _{i=1,\ldots ,N}\mathbb {E}\left[ \left| X_t^{i,\varepsilon }-Y_t^{i,\varepsilon }\right| ^2\right] \le C_1\varepsilon ^{-d}\int _0^t\sup \limits _{i=1,\ldots ,N}\mathbb {E}\left[ \left| X_s^{i,\varepsilon }-Y_s^{i,\varepsilon }\right| ^2\right] \, \mathrm{d} s+\frac{C_2\varepsilon ^{-2(d-1)}}{N-1}. \end{aligned}$$

Applying Gronwall’s inequality further yields that

$$\begin{aligned} \sup \limits _{t\in [0,T]}\sup \limits _{i=1,\ldots ,N}\mathbb {E}\left[ \left| X_t^{i,\varepsilon }-Y_t^i\right| ^2\right] \le \frac{C_2\varepsilon ^{-2(d-1)}}{N-1}e^{C_1\varepsilon ^{-d}T}\le C\frac{(\delta \ln (N))^{\frac{2d-2}{d}} }{N^{1-C\delta }}, \end{aligned}$$

where we let $e^{\varepsilon ^{-d}}\le N^{\delta }$, i.e., $\varepsilon ^{-d}\le \delta \ln (N)$, for any fixed $0<\delta <\frac{1}{C}$. The proof is completed. $\square$

Theorem 5.1 implies the convergence in law of the empirical measure in the following sense:

### Corollary 2

Under the same assumptions as in Theorem 5.1, the empirical measure

5.14 $$\begin{aligned} \rho _t^{\varepsilon ,N}:=\frac{1}{N}\sum _{i=1}^{N}\delta _{X_t^{i,\varepsilon }} \end{aligned}$$

associated with the stochastic particle system (1.5) converges weakly to unique solution $\rho _t$ to the nonlinear SPDE (1.3). More precisely, for any fixed $0<\delta \ll 1$, such that $\varepsilon ^{-d}= \delta \ln (N)$ and $C\delta <1$, it holds that for all $t\in [0,T]$

5.15 $$\begin{aligned} \mathbb {E}\left[ |\langle \rho _t^{\varepsilon ,N},\phi \rangle -\langle \rho _t,\phi \rangle |^2\right] \le C\left( \frac{(\delta \ln (N))^{\frac{2d-2}{d}} }{N^{1-C\delta }}+\frac{1}{N}+(\delta \ln (N))^{-\frac{2}{d}}\right) , \end{aligned}$$

for any $\phi \in C_c^1(\mathbb {R}^d)$, where *C* depends only on $\left\Vert \phi \, \right\Vert _{C^1}$, $\chi ,T,\lambda ,\varLambda ,d$, and $\left\Vert \rho _0 \, \right\Vert _{ W^{2,2}(\mathbb {R}^d)}$.

### Proof

Let us compute

5.16 $$\begin{aligned}&\mathbb {E}\left[ |\langle \rho _t^{\varepsilon ,N},\phi \rangle -\langle \rho _t^\varepsilon ,\phi \rangle |^2\right] \nonumber \\&\quad =\mathbb {E}\left[ \left| \frac{1}{N}\sum _{i=1}^{N}\phi (X_t^{i,\varepsilon })-\int _{\mathbb {R}^d}\phi (x)\rho _t^\varepsilon (x) \, \mathrm{d} x\right| ^2\right] \nonumber \\&\quad \le 2\mathbb {E}\left[ |\phi (X_t^{1,\varepsilon })-\phi (Y_t^{1,\varepsilon })|^2\right] +2\mathbb {E}\left[ \left| \frac{1}{N}\sum _{i=1}^{N}\phi (Y_t^{i,\varepsilon })-\int _{\mathbb {R}^d}\phi (x)\rho _t^\varepsilon (x) \, \mathrm{d} x\right| ^2\right] \nonumber \\&\quad =: I_1+I_2. \end{aligned}$$

According to (5.10), one has

5.17 $$\begin{aligned} I_1\le & {} 2\left\Vert \nabla \phi \, \right\Vert _{\infty }^2\mathbb {E}\left[ |X_t^{1,\varepsilon }-Y_t^{1,\varepsilon }|^2\right] \nonumber \\\le & {} C\frac{(\delta \ln (N))^{\frac{2d-2}{d}} }{N^{1-C\delta }}, \end{aligned}$$

where *C* depends only on $\left\Vert \nabla \phi \, \right\Vert _{\infty }$, $\chi ,T,\lambda ,\varLambda ,d$ and $\left\Vert \rho _0 \, \right\Vert _{W^{2,2}(\mathbb {R}^d)}$. To estimate $I_2$, we compute that

5.18 $$\begin{aligned}&\mathbb {E}\left[ \left| \frac{1}{N}\sum _{i=1}^{N}\phi (Y_t^{i,\varepsilon })-\int _{\mathbb {R}^d}\phi (x)\rho _t^\varepsilon (x) \, \mathrm{d} x\right| ^2\right] \nonumber \\&\quad \le \, \frac{1}{N^2}\sum _{i=1}^{N}\mathbb {E}\left[ \left| \phi (Y_t^{i,\varepsilon })-\int _{\mathbb {R}^d}\phi (x)\rho _t^\varepsilon (x)\right| ^2\right] \le \, C\frac{1}{N}, \end{aligned}$$

where *C* depends only on $\left\Vert \phi \, \right\Vert _{\infty }$. This combined with (5.17) implies

5.19 $$\begin{aligned} \mathbb {E}\left[ |\langle \rho _t^{\varepsilon ,N},\phi \rangle -\langle \rho _t^\varepsilon ,\phi \rangle |^2\right] \le C\left( \frac{(\delta \ln (N))^{\frac{2d-2}{d}} }{N^{1-C\delta }}+\frac{1}{N} \right) , \end{aligned}$$

Next, using (5.9) we compute

5.20 $$\begin{aligned} \mathbb {E}\left[ |\langle \rho _t^{\varepsilon },\phi \rangle -\langle \rho _t,\phi \rangle |^2\right] \le C\mathbb {E}\left[ \sup _{t\in [0,T]}\left\Vert \rho ^\varepsilon _t-\rho _t \, \right\Vert _4^2\right] =C\left\Vert \rho ^\varepsilon -\rho \, \right\Vert _{S_{\mathcal {F}^W}^{2}([0,T];L^4(\mathbb {R}^d))}^2\le C\varepsilon ^2. \end{aligned}$$

Hence, one has

5.21 $$\begin{aligned} \mathbb {E}\left[ |\langle \rho _t^{\varepsilon ,N},\phi \rangle -\langle \rho _t,\phi \rangle |^2\right]&\le 2\mathbb {E}\left[ |\langle \rho _t^{\varepsilon ,N},\phi \rangle -\langle \rho _t^\varepsilon ,\phi \rangle |^2\right] +2\mathbb {E}\left[ |\langle \rho _t^{\varepsilon },\phi \rangle -\langle \rho _t,\phi \rangle |^2\right] \nonumber \\&\le C\left( \frac{(\delta \ln (N))^{\frac{2d-2}{d}} }{N^{1-C\delta }}+\frac{1}{N}+(\delta \ln (N))^{-\frac{2}{d}} \right) . \end{aligned}$$

This completes the proof. $\square$

## Footnotes

1 The dimension of Wiener process *W* may be different from $d'$; we assume the same dimensionality for notational simplicity.

2 Here, the initial condition $\rho _0\in \mathcal {H}^{\frac{1}{2}}_{4}(\mathbb {R}^d)$ is required by the $L^p$-theory of SPDEs (see Krylov 1999, Theorem 5.1) for $p=4$.

3 The selections of $\ell$ and $\kappa$ are not unique; a particular case is to take $\kappa _0\le \frac{1}{\chi \ell }$ with

$$\begin{aligned} \ell = \exp \left\{ \frac{T}{4} \left( \frac{(12\varLambda ^2)^2}{2\lambda }+\frac{(6 S_d)^2}{\lambda }\right) \right\} . \end{aligned}$$

4 The fact $u\in S_{\mathcal {F}^W}^2 ([0,T];L^2(\mathbb {R}^d))$ is not claimed in Du et al. (2011), Zhou (1992), but it follows straightforwardly from Ren et al. (2007, Theorem A.2) for Itô’s formula of square norms. It is similar in the relation (4.6).

## References

[CR1] Bellomo N, Bellouquid A, Chouhad N (2016). From a multiscale derivation of nonlinear cross-diffusion models to Keller–Segel models in a Navier–Stokes fluid. Math. Models Methods Appl. Sci..

[CR2] Bensoussan A, Frehse J, Yam P (2013). Mean Field Games and Mean Field Type Control Theory.

[CR3] Biler P (2018). Mathematical challenges in the theory of chemotaxis. Sci. Ann. Math. Sil..

[CR4] Biler P, Karch G (2010). Blowup of solutions to generalized Keller–Segel model. J. Evol. Equ..

[CR5] Blanchet A, Dolbeault J, Perthame B (2006). Two-dimensional Keller–Segel model: optimal critical mass and qualitative properties of the solutions, 2006. Electron. J. Differ. Equ. (EJDE) [electronic only].

[CR6] Blanchet A, Carrillo JA, Masmoudi N (2008). Infinite time aggregation for the critical Patlak–Keller–Segel model in $$\mathbb{R}^2$$. Commun. Pure Appl. Math..

[CR7] Bresch D, Jabin P-E, Wang Z (2019). On mean-field limits and quantitative estimates with a large class of singular kernels: application to the Patlak–Keller–Segel model. C. R. Math..

[CR8] Brezis H (2010). Functional Analysis. Sobolev Spaces and Partial Differential Equations.

[CR9] Brzezniak Z, Flandoli F, Maurelli M (2016). Existence and uniqueness for stochastic 2D Euler flows with bounded vorticity. Arch. Ration. Mech. Anal..

[CR10] Burini D, Chouhad N (2019). A multiscale view of nonlinear diffusion in biology: from cells to tissues. Math. Models Methods Appl. Sci..

[CR11] Carmona R, Delarue F (2018). Probabilistic Theory of Mean Field Games with Applications I-II.

[CR12] Carmona R, Delarue F, Lacker D (2016). Mean field games with common noise. Ann. Probab..

[CR13] Carrillo JA, Choi Y-P, Hauray M (2014). The derivation of swarming models: mean-field limit and Wasserstein distances. Collective Dyn. Bacteria Crowds.

[CR14] Cattiaux, P., Pédèches, L.: The 2-d stochastic Keller–Segel particle model: existence and uniqueness (2016). arXiv preprint arXiv:1601.08026

[CR15] Choi Y-P, Salem S (2019). Cucker–Smale flocking particles with multiplicative noises: stochastic mean-field limit and phase transition. Kinetic Relat. Models.

[CR16] Coghi M, Flandoli F (2016). Propagation of chaos for interacting particles subject to environmental noise. Ann. Appl. Probab..

[CR17] Corrias L, Perthame B, Zaag H (2004). Global solutions of some chemotaxis and angiogenesis systems in high space dimensions. Milan J. Math..

[CR18] Dolbeault J, Perthame B (2004). Optimal critical mass in the two dimensional Keller–Segel model in $$\mathbb{R}^2$$. C. R. Math..

[CR19] Du K, Qiu J, Tang S (2011). $$L^p$$ theory for super-parabolic backward stochastic partial differential equations in the whole space. Appl. Math. Optim..

[CR20] Escudero C (2006). The fractional Keller–Segel model. Nonlinearity.

[CR21] Fetecau RC, Huang H, Sun W (2019). Propagation of chaos for the Keller–Segel equation over bounded domains. J. Differ. Equ..

[CR22] Fournier N, Jourdain B (2017). Stochastic particle approximation of the Keller–Segel equation and two-dimensional generalization of Bessel processes. Ann. Appl. Probab..

[CR23] Haškovec J, Schmeiser C (2011). Convergence of a stochastic particle approximation for measure solutions of the 2d Keller–Segel system. Commun. Partial Differ. Equ..

[CR24] Hilbert D (1902). Mathematical problems. Bull. Am. Math. Soc..

[CR25] Hillen T, Painter KJ (2009). A user’s guide to PDE models for chemotaxis. J. Math. Biol..

[CR26] Horstmann, D.: From 1970 Until Present: The Keller–Segel Model in Chemotaxis and Its Consequences (2003)

[CR27] Huang H, Liu J-G (2016). Well-posedness for the Keller–Segel equation with fractional Laplacian and the theory of propagation of chaos. Kinet. Relat. Models.

[CR28] Huang H, Liu J-G (2017). Discrete-in-time random particle blob method for the Keller–Segel equation and convergence analysis. Commun. Math. Sci..

[CR29] Huang H, Liu J-G (2017). Error estimate of a random particle blob method for the Keller–Segel equation. Math. Comput..

[CR30] Huang H, Liu J-G, Lu J (2019). Learning interacting particle systems: diffusion parameter estimation for aggregation equations. Math. Models Methods Appl. Sci..

[CR31] Jabin P-E, Wang Z (2017). Mean Field Limit for Stochastic Particle Systems, Active Particles.

[CR32] Jäger W, Luckhaus S (1992). On explosions of solutions to a system of partial differential equations modelling chemotaxis. Trans. Am. Math. Soc..

[CR33] Karatzas I, Shreve SE (1998). Brownian Motion and Stochastic Calculus.

[CR34] Keller EF, Segel LA (1970). Initiation of slime mold aggregation viewed as an instability. J. Theor. Biol..

[CR35] Krylov NV (1999). An analytic approach to SPDEs, Stochastic partial differential equations: six perspectives. AMS Math. Surv. Monogr..

[CR36] Krylov NV (2010). Itô’s formula for the $$L_p$$-norm of stochastic $$W^1_p$$-valued processes. Probab. Theory Relat. Fields.

[CR37] Perthame B (2006). Transport Equations in Biology.

[CR38] Ren J, Röckner M, Wang F (2007). Stochastic generalized porous media and fast diffusion equations. J. Differ. Equ..

[CR39] Triebel H (1983). Theory of Function Spaces.

[CR40] Yang Z, Tang S (2013). Dynkin game of stochastic differential equations with random coefficients, and associated backward stochastic partial differential variational inequality. SIAM J. Control Optim..

[CR41] Zhou X (1992). A duality analysis on stochastic partial differential equations. J. Funct. Anal..

